# Biofilm Source Shapes the Tolerance to Short‐Term Ciprofloxacin Exposure

**DOI:** 10.1111/1758-2229.70408

**Published:** 2026-08-30

**Authors:** Clara Benavent‐Celma, Sophie Oster, Sebastian Pietz, Sabine Filker, Mirco Bundschuh, Timo Mühlhaus, Alexander Feckler

**Affiliations:** ^1^ The James Hutton Institute Aberdeen Scotland; ^2^ Department of Natural and Environmental Sciences, iES Landau, Institute for Environmental Sciences RPTU University Kaiserslautern‐Landau Landau Germany; ^3^ Department of Biology RPTU University Kaiserslautern‐Landau Kaiserslautern Germany

**Keywords:** freshwater ecosystems, microbial diversity, pollution‐induced community tolerance, press disturbance, primary production

## Abstract

Periphytic biofilms are key mediators of freshwater ecosystem processes but are increasingly exposed to anthropogenic stressors. We compared biofilms sampled upstream and downstream of a wastewater treatment plant (WWTP) effluent, two source communities presumed to differ in prior exposure to wastewater‐associated press disturbance, to examine differences in tolerance to ciprofloxacin (CIP) exposure. We assessed biomass, photosynthetic pigments and fatty acid (FA) profiles as well as microbial community structure using qPCR and metabarcoding. Downstream‐sourced biofilms exhibited higher biomass stability, reduced pigment and FA losses and fewer bacterial and eukaryotic community shifts under CIP exposure than upstream‐sourced biofilms. Upstream‐sourced communities, despite higher baseline pigment and FA contents, showed pronounced sensitivity to CIP, including biomass declines and reduced prokaryotic diversity. Taxon‐specific responses revealed stress‐tolerant *Chlorophyceae*, cyanobacteria and heterotrophic taxa, which may have contributed to higher functional stability under the tested conditions. Our findings show that biofilm responses to CIP varied with source site and community composition. While not a direct test of environmental filtering, the observed patterns are consistent with its predictions. The results further suggest that legacy effects and spatial heterogeneity may influence biofilm vulnerability to antibiotic exposure, although this requires validation across multiple WWTP‐impacted and reference sites.

## Graphical abstract

Biofilms sourced downstream of a wastewater treatment plant showed greater tolerance to ciprofloxacin than upstream‐sourced communities. The results suggest that environmental history and community composition influence freshwater biofilm responses to antibiotic stress.

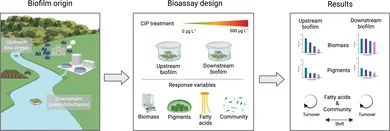

## 1 Introduction

Periphytic biofilms are complex communities comprising autotrophic and heterotrophic taxa, including algae, bacteria, fungi and protists, which colonise submerged surfaces (Lamprecht et al. 2022; Wetzel 1983). These biofilms play a crucial role in ecosystem functioning, contributing substantially to primary production, nutrient cycling and water quality improvement in surface waters (Battin et al. 2016; Miao et al. 2019). By converting inorganic carbon into organic forms, periphytic biofilms support higher trophic levels, including invertebrates and fish, by serving as a primary food source (Vannote et al. 1980). However, the presence of chemical pollutants, such as pharmaceuticals, in aquatic ecosystems can have severe effects on biofilm composition (Oster 2025) and functioning (Feckler et al. 2025), with potential cascading effects on the entire food web (Konschak et al. 2020; Meyer et al. 2016).

Pharmaceutical pollution in aquatic ecosystems stems from their widespread application in human and veterinary medicine, disease prophylaxis and treatment (Bernhardt et al. 2017; Łukaszewicz et al. 2017; Richmond et al. 2018), as well as growth promotion (Visek 1978) and crop protection (González‐Pleiter et al. 2013). Due to incomplete metabolisation and degradation in wastewater treatment plants (WWTPs), significant amounts of pharmaceuticals are frequently detected in WWTP effluents (Tlili et al. 2017; Rodriguez‐mozaz et al. 2014). As a result, WWTP effluents represent a constant and significant source of complex pharmaceutical mixtures to freshwater ecosystems, shaping the community composition of biofilms through environmental filtering (Tlili et al. 2017; Cadotte and Tucker 2017; Marizzi del Olmo et al. 2025). Among these, antibiotics act as strong selective agents in aquatic ecosystems, filtering for tolerant microbial assemblages (Wangberg 1988; Vinebrooke et al. 2004). Owing to their specific modes of action, antibiotics directly affect bacteria, including cyanobacteria. Some antibiotic classes, including fluoroquinolones such as ciprofloxacin (CIP), can also affect eukaryotic algae through conserved molecular targets within chloroplasts, leading to shifts in community composition with potential implications for primary production and food‐web dynamics (Grenni et al. 2018; Kümmerer 2009; González‐Pleiter et al. 2013; Nie et al. 2009).

Here we compare periphytic biofilm communities sampled upstream and downstream of a WWTP effluent to examine whether their presumed difference in wastewater exposure history is associated with differences in tolerance to antibiotic stress. Biofilms were cultured under controlled laboratory conditions and subsequently exposed to a gradient of CIP, an antibiotic selected due to its widespread use and frequent occurrence in aquatic environments (Al‐buriahi et al. 2022; Booth et al. 2020; Fick et al. 2009). Structural responses were evaluated using taxonomic and community composition data, while functional responses were approximated using total biomass accrual, an established proxy for net primary production and community‐level productivity in periphytic biofilms (Steinman et al. 2006; Porsbring et al. 2007), alongside pigment composition and FA profiles as proxies for phototrophic activity and nutritional quality, respectively (Feckler et al. 2025; Pietz et al. 2023). We did not directly measure metabolic endpoints such as respiration or gross primary production; wherever we refer to ‘function’ or ‘functional responses’ in this manuscript, we refer specifically to the biomass‐based proxies. Community composition and taxonomic shifts (algae, diatoms, bacteria, fungi) were further resolved by metabarcoding and qPCR, respectively (Fierer et al. 2005; Rivera et al. 2023). Because our design compares only one upstream and one downstream site within a single river system, differences between source communities cannot be attributed solely to WWTP‐derived disturbances and may reflect other spatially structured environmental factors (e.g., hydrology, nutrient status and light availability). We therefore use ‘source site’ to denote the two sampled communities rather than a replicated test of WWTP influence. Following a 28‐day common‐garden incubation under identical laboratory conditions, biofilms exposed to CIP were developed from upstream‐ and downstream‐sourced inocula rather than being native field biofilms. We therefore refer to them as upstream‐sourced and downstream‐sourced biofilms. We hypothesised that biofilms grown from downstream‐sourced communities, presumed to reflect prior chronic exposure to WWTP effluents, would exhibit greater tolerance to CIP and lower structural and functional shifts upon CIP exposure compared to upstream‐sourced communities (Gallagher and Reisinger 2020; Ahmed et al. 2018). Specifically, we expected higher tolerance being reflected in lower changes in total biomass, pigment and FA content, as well as reduced prokaryotic and eukaryotic community turnover, consistent with selection for stress‐tolerant taxa under sustained effluent pressure (Uruén et al. 2021).

## 2 Experimental Procedures

### 2.1 Biofilm Colonisation and CIP Exposure

Biofilm sampling was conducted at the Queich river, Germany (49°12′09.7″ N 8°11′17.6″ E), approximately 250 m up‐ and downstream of a municipal WWTP effluent. The municipal WWTP in Landau (Rhineland‐Palatinate, Germany) receives and treats wastewater from approximately 51,000 residents, with a treatment capacity of up to 80,000 population equivalents, reflecting domestic, agricultural and industrial sources. Treated effluent is discharged into the Queich river with varying discharge rates (0.5–1.3 m^3^ s^−1^), influenced by seasonal changes and weather conditions (Sturm et al. 2022; Wasser 3.0). At each sampling site, submersed cobbles (~20 cm in diameter) were collected in February 2024 and transported to the laboratory in river water matching the respective sampling sites. Biofilm from each of the sampling sites was scraped from 10 biofilm‐covered cobbles using sterile toothbrushes and subsequently passed through a 50‐μm sieve to remove invertebrates (e.g., chironomids), soil particles and plant debris. The filtrate was diluted with 2 L of river water matching the sampling site, which had also been passed through a 50‐μm sieve. The inoculum was homogenised overnight on a magnetic stirrer at 200 rpm. On the following day, 1 L of the biofilm inoculum from each sampling site was added to separate plastic containers, each containing 80 unglazed ceramic tiles (4.7 × 4.7 cm), which had been sterilised at 250°C for 5 h, along with 20 L of artificial medium (Borgmann 1996, Rybicki and Jungmann 2018, Table S1). The medium was renewed every 7 days over a 28‐days period. Colonisation was conducted in a climate‐controlled room set at 20°C ± 1°C and a 16:8 h light: dark rhythm. The intensity of the photosynthetically active radiation (PAR) corresponded to the irradiance on streambeds during summer months (~40 μmol m^−2^ s^−1^ PAR; Hill and Dimick 2002).

Following the 28‐days lasting colonisation period, tiles were exposed to CIP (0, 5, 50 and 500 μg L^−1^; nominal concentrations) for 14 days in crystallising dishes, with each concentration being replicated five times per sampling site. Each crystallising dish was filled with 200 mL medium (Table S1) and four randomly selected tiles overgrown by biofilm from one of the respective sampling sites. The medium with its CIP concentrations was renewed after 7 days to ensure constant availability of nutrients and exposure to CIP. The experiments were conducted under the same conditions as detailed above. After 14 days, biofilm was scraped from tiles using sterile surgical blades, transferred into 15‐mL centrifuge tubes and freeze dried (−55°C; Christ Alpha 1–4 LSCbasic) for 48 h. Water samples for the verification of CIP concentrations were taken approximately 30 min after the test setup and just before the water exchange (i.e., after 7 days of exposure) and stored at −20°C until analysis as per Oster (2025). Measured CIP concentrations closely matched nominal concentrations at the start of each exposure interval (Table 1). Concentrations declined over the following 7 days, particularly at higher treatment levels, before medium renewal restored them to near‐nominal levels. This decline likely reflects a combination of sorption, biological uptake and degradation processes. To account for the decline in CIP concentrations over time, we calculated and report linear time‐weighted average concentrations in the following table. These calculations revealed time‐weighted average concentrations of approximately 0, 3, 31 and 310 μg L^−1^ (Table 1), which still accounts for the spacing factor of 10 that we aimed for.

**TABLE 1:** Nominal and measured (mean ± standard deviation) ciprofloxacin concentrations at the time of test setup (0 day) and just before the water exchange (7 days).

Nominal concentration (μg L^−1^)	Time (d)	Measured concentration (μg L^−1^)	Linear time‐weighted average (μg L^−1^)
0	0	< LOD	< LOD
	7	< LOD	
5	0	3.20 ± 0.50	2.8
	7	2.33 ± 0.79	
50	0	50.6 ± 5.6	30.5
	7	10.4 ± 7.8	
500	0	528.0 ± 190.0	310.0
	7	91.4 ± 17.3	

### 2.2 Biofilm Biomass Estimation

After freeze drying, the biofilm was weighed to the nearest 0.01 mg and the biomass was normalised as mg cm^−2^. Afterwards, the biofilm from each replicate was aliquoted into Eppendorf tubes (~10 mg for pigment analyses, ~10 mg for the quantification of phospholipid FA and ~30 mg for DNA extraction and subsequent molecular analyses) and stored at −20°C until analysis.

### 2.3 Pigment Analyses

Pigment analyses were performed following Jeffrey and Humphrey (1975) and Lauridsen et al. (2011), however, adapted for the use of a microplate reader (Feckler et al. 2018). Briefly, pigments were extracted from approximately 10 mg of biofilm in 20‐mL glass scintillation vials using 3 mL 90% acetone (analytical grade). Vials were fully covered in aluminium foil to prevent pigment photodegradation and stored at −20°C for 24 h. Subsequently, samples were sonicated for 2 min, centrifuged for 15 min at 3500 rpm to sediment particles and passed through syringe filters (pore size 0.45 μm; PP; Thermo Scientific). From each extract, three technical replicates of 300 μL were transferred to 96‐microwell plates (Corning 96‐well Solid Black; Corning, NY, USA). Absorbance was measured in the range of 350–700 nm (Tecan Infinite 200 PRO; Tecan Group, Männedorf, Switzerland) and pigment concentrations were calculated following formulas in Jeffrey and Humphrey (1975) for mixed phytoplankton populations containing chlorophylls *a*, *b* and *c*s (sum of c_1_ and c_2_). The results from the technical replicates were averaged, extrapolated to the total sample volume and normalised to the weight of the biofilm aliquot used for pigment analysis.

### 2.4 Fatty Acid Analysis

Fatty acids (FAs) were analysed following protocols published in Fink (2013) and Pietz et al. (2023). Briefly, lipids were extracted from freeze‐dried samples of known dry weight (~10 mg) using 5 mL of a dichloromethane: methanol mixture (2:1; v:v) supplemented with 100 μL of triacylglycerol containing three deuterated 18:0 FAs (Tristearin‐D105; Larodan) as an internal standard (Konschak et al. 2020). Samples were incubated at −20°C overnight, vortexed and the extracts were dried under a nitrogen stream at 40°C. Lipid hydrolysis and subsequent methylation of FAs to fatty acid methyl esters (FAMEs) was achieved by adding 5 mL of 3 N methanolic HCl (Sigma‐Aldrich) and incubating the samples at 70°C for 20 min. FAMEs were extracted with isohexane, evaporated under a nitrogen stream at 40°C and finally dissolved in 100 μL of dichloromethane for storage at −20°C until analysis.

Gas chromatography (GC) with flame‐ionisation detection (Trace GC Ultra; Thermo Fisher Scientific) was performed using a Restek FAMEWAX capillary GC column (30 m × 0.25 mm, 0.25 μm film thickness) and helium (1.4 mL min^−1^) as carrier gas. FAMEs were identified based on their retention times using FAME standards (37‐component FAME Mix; Supelco CRM47885) and quantified (μg FA mL^−1^) using an external standard calibration. The FAMEs of the internal standard (Methyl D‐35 Octadecanoate; Larodan) were added to the calibration standards at the same concentration as the lipid internal standard (Tristearin‐D105). FA concentrations were adjusted for the recovery rate of the internal standard and blank values. The corrected FA concentrations were then normalised to the dry weight of each sample (μg FA mg dry weight^−1^).

### 2.5 Molecular Biofilm Analyses

DNA was extracted from freeze‐dried samples (~30 mg) using the DNeasy PowerBiofilm extraction kit (Qiagen, Hilden, Germany) following the manufacturer's instructions. The DNA amount in each extract was quantified using a Qubit fluorimeter (Invitrogen, CA, USA) and its quality was checked using a Nanodrop ND‐1000 spectrophotometer (peqlab, Erlangen, Germany). We included extraction controls to account for potential cross‐contamination. Extraction blanks consistently returned negative results, confirming the absence of cross‐contamination.

### 2.6 Quantification of Microbial Abundances

Operon copy numbers of bacteria, fungi, green algae and diatoms were quantified as proxies for microbial abundances using the protocols described in Manerkar et al. (2014) and Feckler et al. (2023). The following primer pairs were used: E8F/E533R for bacteria (Baker et al. 2003), ITS3F/ITS4R for fungi (White et al. 1990), EUK528f/CHLO02r for green algae (Elwood et al. 1985; Simon et al. 2000) and 1209f/18SR1 for diatoms (Godhe et al. 2008). Pure DNA extracts were used in group‐specific SYBR Green qPCR reactions (Thermo Fisher Scientific GmbH, Dreieich, Germany). Calibration curves were generated using model amplicons of *Escherichia coli* (Migula 1895) (Castellani and Chalmers 1919) (bacteria), *Tetracladium marchalianum* De Wild. (fungi), *Desmodesmus subspicatus* (Chodat) Hegewald et Schmidt (green algae) and *Nitzschia palea* (Kütz.) Kuntze 1898 (diatoms), covering a range of 10^3^ to 10^9^ operon copies (Table S2). Melting curve analyses were performed at the end of each qPCR run to verify the specificity of the assays. Detailed description of the respective assays, reaction compositions, cycling conditions and data analysis can be found in the underlying studies (Manerkar et al. 2014; Feckler et al. 2023). All qPCR reactions were performed on a Mastercycler ep gradient S (Eppendorf, Hamburg, Germany) using 0.2‐mL 8‐tube strips covered with clear optical 8‐cap strips (Sarstedt AG & Co. KG, Nümbrecht, Germany). All results were normalised to the respective dry weight of the samples (operon copies mg dry weight^−1^).

### 2.7 DNA Metabarcoding, Sequence Data Processing and Taxonomic Assignment

For metabarcoding, 50 ng of DNA was used to amplify the hypervariable V4‐region of the microeukaryotic 18S and prokaryotic 16S rRNA gene using the primer sets V4‐TAR‐EUK_F (5′‐CCAGCASCYGCGGTAATTCC‐3′) and V4‐TAR‐Euk_R (5′‐CAGACTTTCGTTCTTGATYRA‐3′) (Stoeck et al. 2010) and 515Fm (5′‐GTGYCAGCMGCCGCGGTAA‐3′) and 806Rm (5′‐GGACTACNVGGGTWTCTAAT‐3′) (Walters et al. 2015), respectively. Both PCR protocols started with an activation step at 98°C for 30 s, followed by 26 cycles consisting of 98°C for 10 s, 63°C for 30 s and 72°C for 30 s and a final extension for 5 min at 72°C. To minimise amplification bias, three independent PCR amplifications were performed per sample and subsequently pooled prior to library preparation. Libraries were constructed using the NEBNext Ultra DNA Library Prep Kit for Illumina (New England Biolabs, USA) following the manufacturer's protocol. Library quality and fragment size distribution were assessed using the Agilent 2100 Bioanalyzer system. Paired‐end sequencing (2 × 250 bp) was carried out on an Illumina MiSeq platform by StarSeq (Mainz, Germany).

Raw reads were initially processed by trimming excessive primer overhangs using *cutadapt v5.0* (Martin 2011). Subsequent read processing was performed with *DADA2 v1.16* (Callahan et al. 2016), applying the filterAndTrim function with parameters truncLen = 230 and maxEE = 1. The truncation length was chosen based on the sequence position where the Phred quality score reached ≥ 30 (Q3) for at least 51% of the reads in the dataset (indicating a base call accuracy of 99.9%; Ewing and Green 1998). Reads were subsequently merged using a 20‐base pair overlap, allowing a maximum of 2 mismatches. Chimera identification and removal were conducted using *vsearch v2.29* (Rognes et al. 2016). Taxonomic classification of the resulting amplicon sequence variants (ASVs) was performed using the SINTAX algorithm (Edgar 2016) against the SILVA database v138.2 (Quast et al. 2013) for prokaryotes and the PR2 database for eukaryotes (Guillou et al. 2013). After merging the ASV‐contingency table with taxonomic annotations, ASVs lacking taxonomic assignment, those occurring only in a single sample (potentially artefactual sequences; Bokulich et al. 2013) and non‐target ASVs (i.e., metazoa and embryophyta) were excluded from further analyses. Raw sequence reads are deposited at NCBI's Sequence Read Archive under accession number PRJNA1438052.

### 2.8 Statistical Analysis

In a Bayesian setting, simple linear models (excluding random effects) were used on univariate data (biomass, pigment and FA content, alpha diversity and microbial abundances) to assess how the source site and CIP exposure impacted the biofilm responses. These models were constructed using the R package *brms* (version 2.22.0) that utilises *rstan* (version 2.32.6; Stan Development Team 2024), running on 4 chains each with 20,000 iterations, a thinning rate of 10 and a warmup period of 16,000 (Bürkner 2017). We employed weakly informative priors for all model coefficients and a normal distribution for the errors following a visual examination of the data. The priors were designed to be informative enough to guide the convergence of the chains and achieve a suitable fit of the models, while remaining sufficiently vague to avoid influencing their results. The assessments of posterior distributions were carried out using the maximum a posteriori estimate (MAP) and a 95% highest‐density credible interval. The posterior odds of negative and positive values are denoted as the Bayes factor (BF) and represent the likelihood ratio of H_1_ (indicating a difference between treatments) to H_0_ (indicating no difference between treatments), based on the available data. The benchmark factor was represented as greater than 1, independent of the outcome's direction, to enhance readability and facilitate comparisons. When all comparisons favour H_1_ over H_0_, the Bayes Factor approaches infinite (i.e., Inf).

Nonmetric multidimensional scaling (NMDS) ordination plots were created to visualise dissimilarities in FA compositions among CIP treatments and source site (Clarke 1993), using absolute amounts of individual FAs and Euclidean distances. Canonical correspondence analysis (CCA) was used to examine the relationships between environmental variables (CIP treatments and source site) and microbial community composition, facilitating the identification of potential drivers of microbial community variation. CCA was selected based on the assumption of unimodal species responses along environmental gradients. Permutational multivariate analysis of variance (PERMANOVA) was conducted to test the statistical significance of differences in microbial community structure and FA composition among CIP treatments, source sites and their interaction. NMDS, CCA and PERMANOVAs were performed using the respective functions in the R package *vegan* (Oksanen et al. 2022). For statistical analyses and graphics, the open‐source software R (R Core Team 2025) was used along add‐on packages. Data, metadata and code are freely available at Zenodo (Benavent‐Celma et al. 2026; https://doi.org/10.5281/zenodo.20355858), largely following the FAIR principles.

## 3 Results

### 3.1 Effects of Source Site on Biofilm Community Structure and Function

**FIGURE 1:**
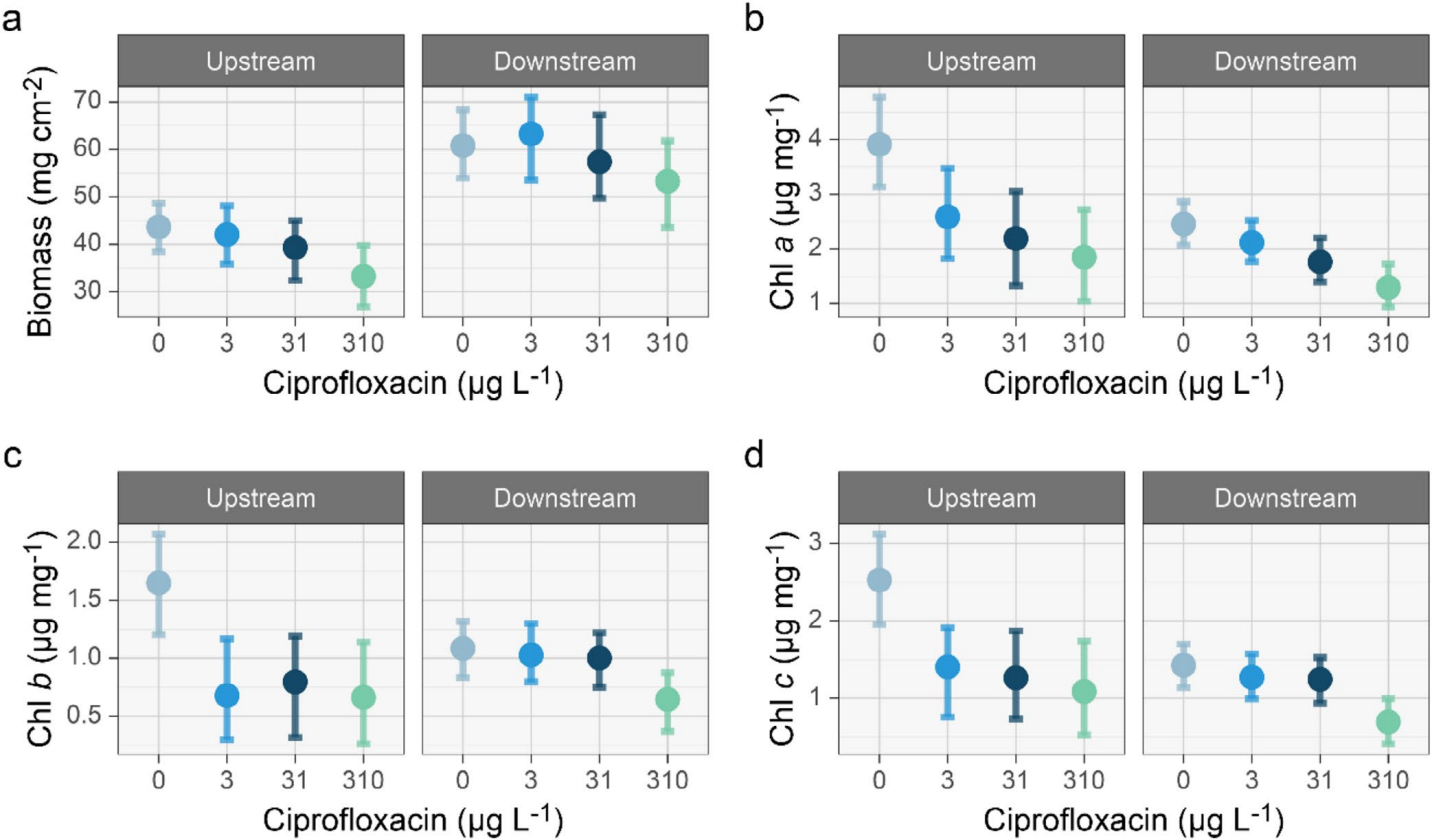
Medians ±95% credible intervals (*n* = 5) of biofilm (a) biomass (mg cm^−2^), (b) chlorophyll *a* (μg mg^−1^), (c) chlorophyll *b* (μg mg^−1^) and (d) chlorophyll *c*s (μg mg^−1^). Colours indicate CIP treatments: 0 μg L^−1^ (control; cyan‐blue), 3 μg L^−1^ (light blue), 31 μg L^−1^ (dark blue), 310 μg L^−1^ (light green). Results are separated by the biofilm source (upstream and downstream). Statistical differences were assessed via Bayesian linear models.

**FIGURE 2:**
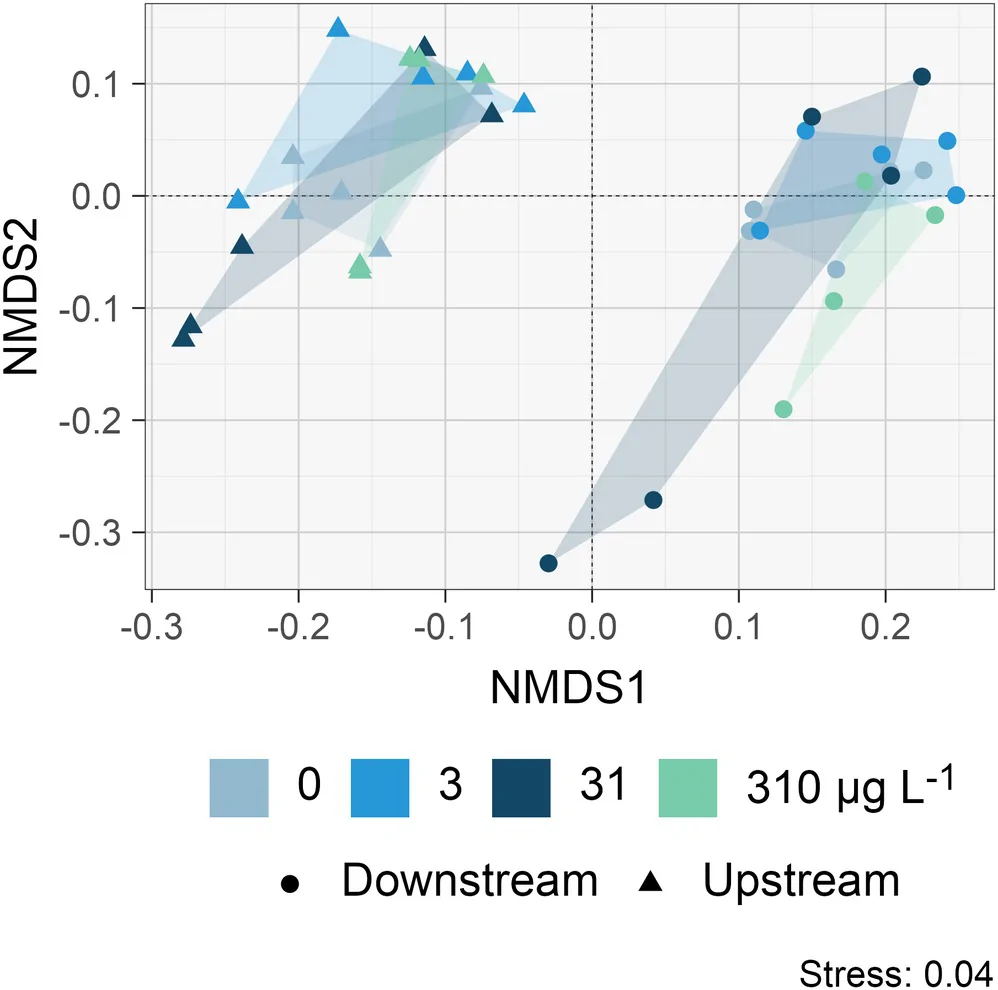
Non‐metric multidimensional scaling (NMDS) ordination of fatty acid (FA) profiles in biofilms originating from the upstream‐sourced (triangles) and downstream‐sourced (circles) sites (*n* = 5). Spider webs connect the replicates. Colours indicate CIP treatments: 0 μg L^−1^ (control; cyan‐blue), 3 μg L^−1^ (light blue), 31 μg L^−1^ (dark blue), 310 μg L^−1^ (light green). The NMDS stress value was below 0.2, indicating a reasonable fit of the data (Clarke 1993).

**FIGURE 3:**
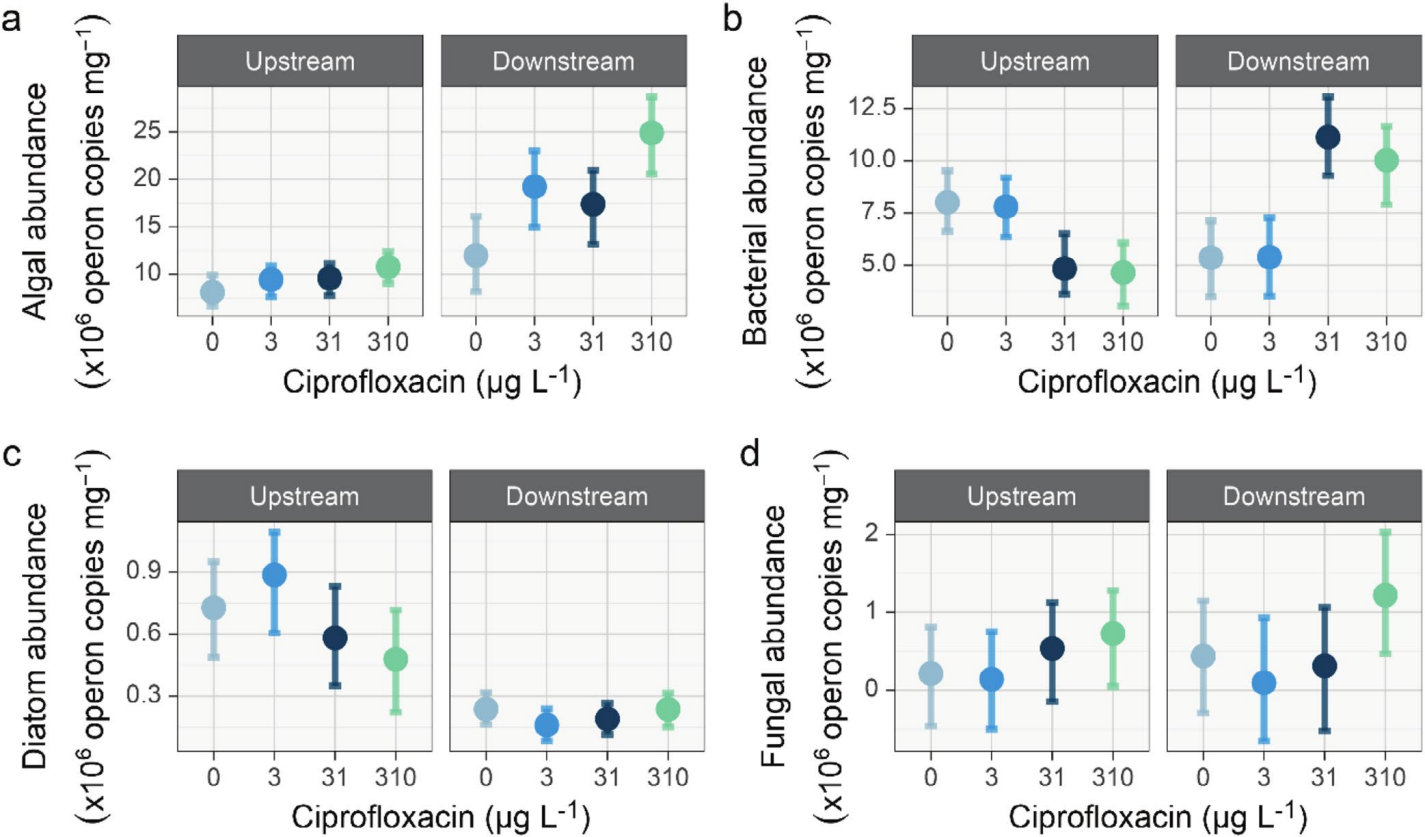
Medians ±95% credible intervals (*n* = 5) of biofilm (a) algal abundance (× 10^6^ operon copies mg^−1^), (b) bacterial abundance (× 10^6^ operon copies mg^−1^), (c) diatom abundance (× 10^6^ operon copies mg^−1^) and (d) fungal abundance (× 10^6^ operon copies mg^−1^). Colours indicate CIP treatments: 0 μg L^−1^ (control; cyan‐blue), 3 μg L^−1^ (light blue), 31 μg L^−1^ (dark blue), 310 μg L^−1^ (light green). Results are separated by the biofilm source (upstream and downstream). Statistical differences were assessed via Bayesian linear models.

For brevity, biofilms grown from upstream‐ and downstream‐sourced inocula are hereafter referred to as ‘upstream‐sourced’ and ‘downstream‐sourced’ biofilms or simply ‘upstream’ and ‘downstream’ in Figures and where unambiguous; these terms describe the source site community rather than measurements made directly on native, field‐collected biofilms. ‘Function’ refers throughout to biomass accrual as a proxy for net primary production (see Introduction); no direct metabolic measurements (e.g., respiration) were taken. In the absence of CIP, downstream‐sourced biofilms exhibited ~35% higher biomass than upstream‐sourced communities (BF = Inf; Figure 1a). In contrast, chlorophyll *a*, *b* and *c*s were ~1.5‐fold higher upstream‐sourced (BF = 79‐Inf; Figure 1b–d, Figure S1b–d). Similarly, saturated (SAFA), monounsaturated (MUFA), polyunsaturated (PUFA) and Ω3 fatty acids were up to four‐fold higher upstream‐sourced (BF = 6.5‐Inf; Table S3). Biofilm source site explained 51% of the variation in FA composition (PERMANOVA, *p* < 0.001; Figure 2, Table S4). Algal abundance was by ~85% higher downstream‐sourced (BF = 2), whereas bacteria (by ~25%) and diatoms (by ~100%) were more abundant upstream‐sourced (BF = 2–36, Figure 3b,c). Fungal abundance was comparable between sites (BF = 1; Figure 3d, Figure S2). Both eukaryotic (Shannon BF = 7; Simpson BF = 1) and prokaryotic (Shannon BF = 12; Simpson BF = 7) alpha diversity tended to be lower downstream‐sourced (Figure S3).

Eukaryotic communities were dominated by *Chlorophyceae*, *Ochrophyta* (mainly diatoms) and fungi, with site‐specific differences: *Chlorophyceae* were more abundant downstream‐sourced (56% vs. 44%), whereas *Ochrophyta* were enriched upstream‐sourced (25% vs. 15%); fungal contributions were similar (~10%–11%; Figure 5a). Downstream‐sourced biofilms were characterised by higher abundances of *Uronema*, *Stigeoclonium* (*Chlorophyceae*) and *Chytridiomycetes*, whereas upstream‐sourced communities were enriched in *Melosira* (Figure 6a). These differences were reflected by CCA, with CCA1 explaining 37% of the variation between sites. Prokaryotic communities were dominated by *Proteobacteria*, *Cyanobacteria* and *Bacteroidetes* and were broadly similar between sites; however, *Cyanobacteria* were more abundant downstream‐sourced (30% vs. 21%), largely driven by *Leptolyngbya* (Figure 5b, Figure 6b).

### 3.2 Effects of CIP on Biofilm Community Structure and Function

CIP induced concentration‐dependent and site‐specific effects on biofilm structure and function. At 3 and 31 μg L^−1^, biomass reductions were minor (< 10%; BF = 1.6–7.7) irrespective of source site, whereas 310 μg L^−1^ reduced biomass by 25% upstream‐sourced (BF = 105.7) and 15% downstream‐sourced (BF = 24.8; Figure 1a, Figure S1a). Chlorophyll *a* declined dose‐dependent in both communities (up to 53%; BF = 4.6–Inf). In contrast, chlorophyll *b* and *c*s were consistently reduced in upstream‐source biofilms (≥ 60% across all concentrations; BF ≥ 199) but decreased downstream‐sourced only at 310 μg L^−1^ (50%; BF = 88), with negligible effects at lower concentrations (BF = 1–5; Figure 1b,c, Figure S1b,c). All FA groups declined with increasing CIP, while stronger responses were observed upstream‐sourced. At 310 μg L^−1^, SAFAs decreased by 35% (BF = 16.8), MUFAs by 42% (BF = 99) and PUFAs and Ω3 FAs by up to 37% (BF = 7.21), whereas effect sizes were generally similar downstream‐sourced (≤ 37%; BF = 1–4; Table S3, Table S5). CIP treatment significantly influenced FA composition, explaining only 8% of total variation (*p* = 0.045; Figure 2, Tables S4 and S5).

**FIGURE 4:**
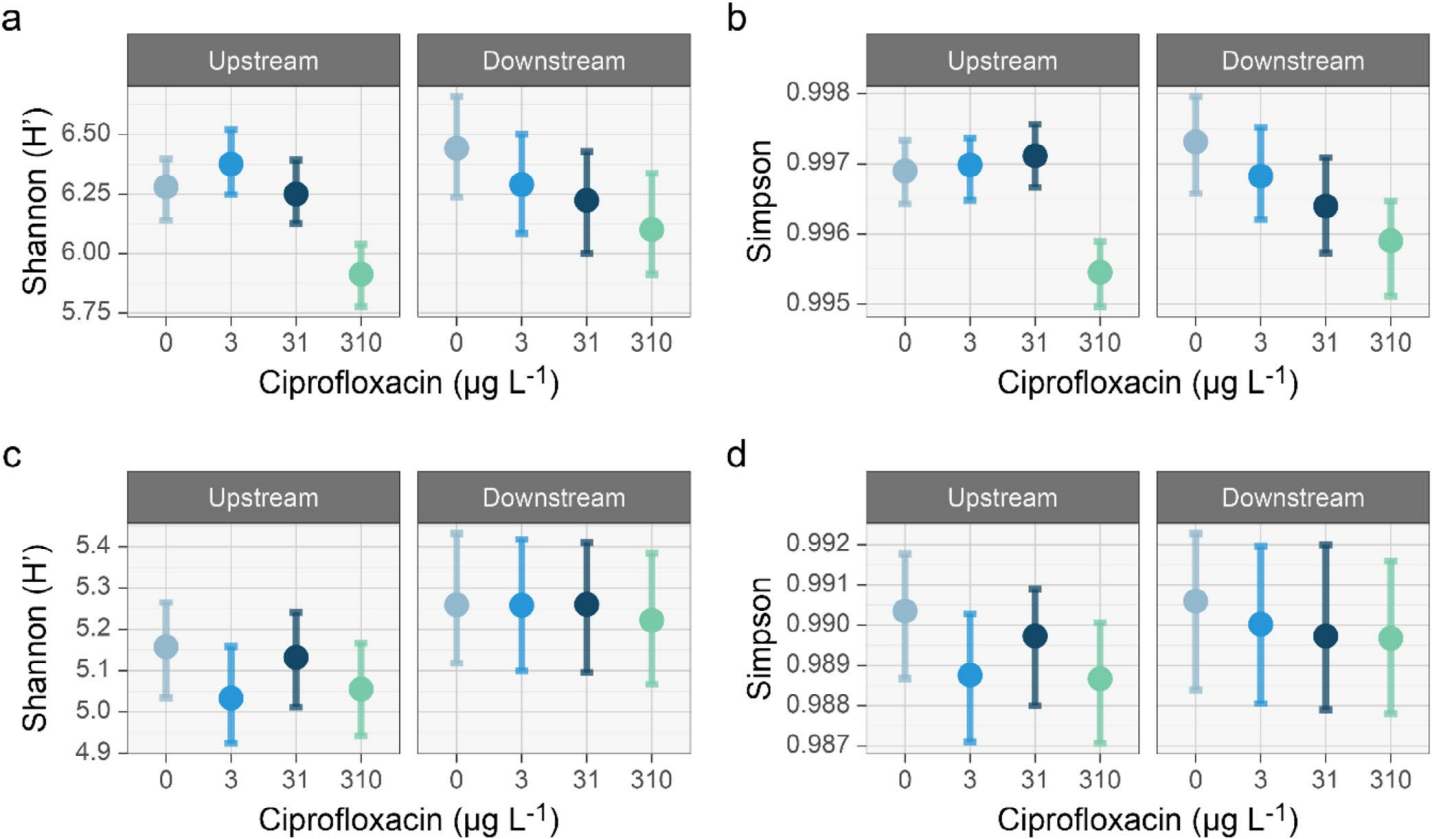
Medians ±95% credible intervals (*n* = 5) of alpha diversity of biofilm communities expressed as the Shannon (H′) and Simpson indices for prokaryotic (a, b) and eukaryotic (c, d) biofilm communities. Colours indicate CIP treatments, namely 0 μg L^−1^ (control; cyan‐blue), 3 μg L^−1^ (light blue), 31 μg L^−1^ (dark blue), 310 μg L^−1^ (light green). Results are separated by the biofilm source (upstream and downstream). Statistical differences were assessed via Bayesian linear models.

**FIGURE 5:**
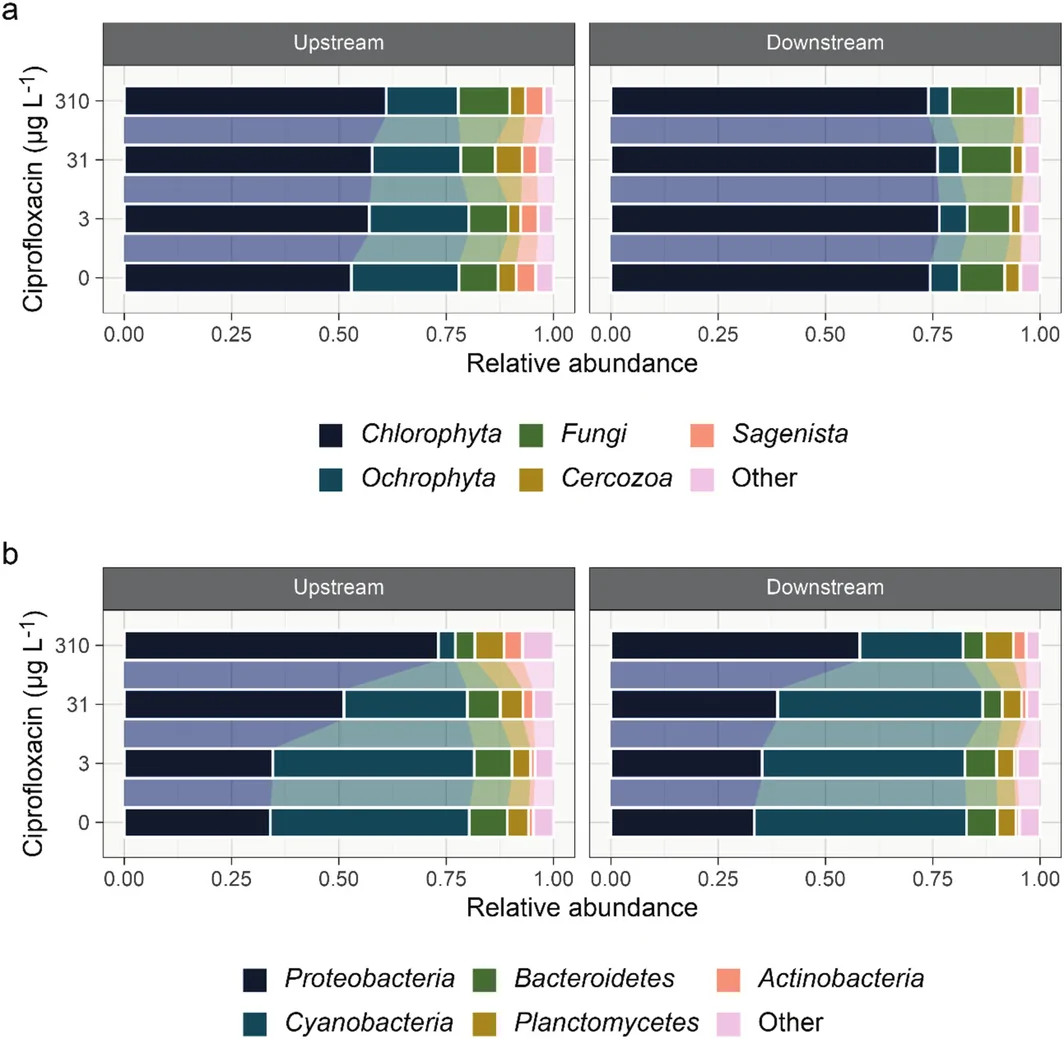
Taxonomic composition of biofilm communities (*n* = 5) for eukaryotes (a) and prokaryotes (b). Stacked bar plots show the relative abundance of microbial taxa at the phylum level at each ciprofloxacin (CIP) concentration. Relative abundances were derived from 18S and 16S rRNA gene sequencing data, respectively, and normalised for sequencing depth. Only the top 5 phyla are shown (Yeoh et al. 2016; Yeoh, Sekiguchi, et al. 2016).

**FIGURE 6:**
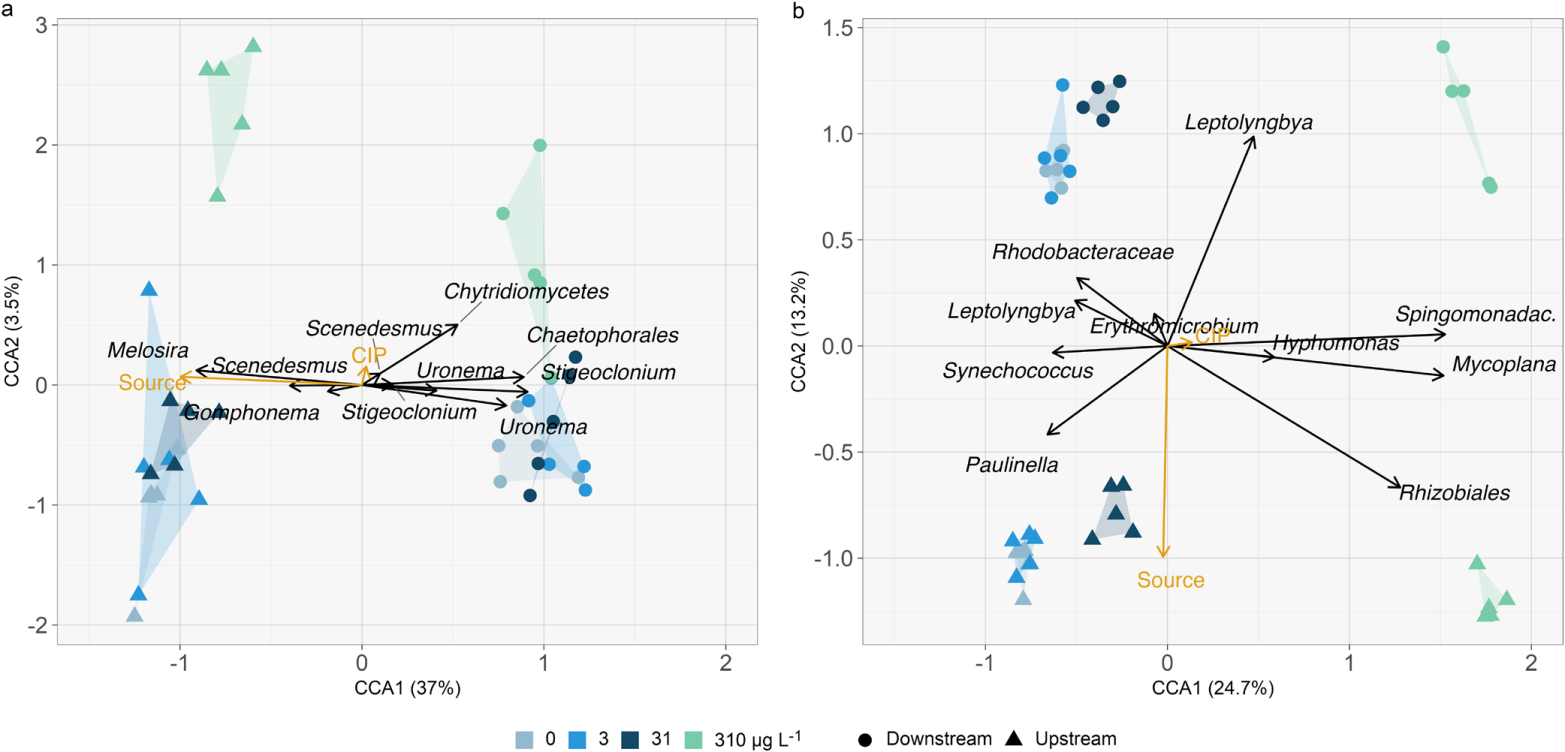
Canonical correspondence analysis (CCA) based on Jaccard similarity illustrating variation in eukaryotic (a) and prokaryotic (b) community composition at the ASV level across upstream‐sourced (triangles) and downstream‐sourced (circles) samples exposed to CIP (*n* = 5). Colours indicate CIP treatments: 0 μg L^−1^ (control; cyan‐blue), 3 μg L^−1^ (light blue), 31 μg L^−1^ (dark blue), 310 μg L^−1^ (light green). Black arrows represent taxa contributing most strongly to the observed variation, highlighting differential abundance patterns associated with source site and CIP exposure.

CIP exposure increased algal abundance across treatments, more markedly downstream‐sourced (45%–108%; BF = 99‐Inf) than upstream‐sourced (16%–33%; BF = 5.2–52.3; Figure 3a, Figure S2a). Diatoms exhibited a unimodal response upstream‐sourced (20% increase at 3 μg L^−1^, followed by ≥ 20% declines at higher concentrations; BF = 4.3–12.3) but remained largely unaffected downstream‐sourced. Bacterial abundance declined strongly upstream‐sourced (−40% at 31 and 310 μg L^−1^; BF = 199–399), whereas it increased downstream‐sourced (≥ 82%; BF = 799‐Inf). Fungal abundance was largely unaffected irrespective of site (Figure 3b, Figure S2b). Eukaryotic alpha diversity remained stable at both sites (Shannon and Simpson BF ≤ 17), whereas prokaryotic diversity declined markedly with increasing CIP upstream‐sourced (BF up to 1599) and downstream‐sourced (Shannon BF = 5–48; Simpson BF = 6–1599; Figure 4). Canonical correspondence analysis indicated modest compositional shifts, with CCA2 explaining 3.5% of the variation associated with CIP exposure (Figure 6b). Taxonomically, CIP increased the relative abundance of *Chlorophyceae* (upstream‐sourced: ≥ 44%; downstream‐sourced: 56%–61%) and *Proteobacteria* (upstream‐sourced: 44%–58%; downstream‐sourced: 40%–50%), while reducing *Ochrophyta* and *Cyanobacteria* (upstream‐sourced: ≥ 8%; downstream‐sourced: ≥ 23%), particularly *Synechococcus*, *Paulinella* and *Leptolyngbya* (Figures 5b and 6b).

### 3.3 Combined Effects of Source Site and CIP on Biofilm Community Structure and Function

Across structural and functional endpoints, biofilm source site was the primary determinant of community composition, consistently reflected in biomass, pigment content, taxa abundances, FA profiles and alpha diversity. CIP imposed additional, site‐specific effects, most evident in upstream‐sourced biofilms, where it reduced biomass, bacterial abundance and prokaryotic diversity and induced moderate shifts in taxonomic composition and FA patterns.

PERMANOVA confirmed the dominant role of source site, explaining 26.5% (*p* = 0.001) and 26.7% (*p* = 0.001) of the variation in eukaryotic and prokaryotic communities, respectively. CIP explained a smaller fraction of variation overall (eukaryotes: 2.6%, *p* = 0.122; prokaryotes: 37.2%, *p* = 0.001). When analysed separately by site, CIP significantly affected upstream‐sourced eukaryotic communities (19.7% variation explained; *p* = 0.002), but not downstream‐sourced communities (5.2%; *p* = 0.478). Canonical correspondence analysis supported these findings. For eukaryotes, CCA1 (37% of variation) separated upstream‐sourced biofilms, associated with *Ochrophyta* (mainly diatoms, e.g., *Melosira*), from downstream‐sourced biofilms, characterised by *Chlorophyceae* (e.g., *Uronema*, *Stigeoclonium*) and *Chytridiomycetes*. CCA2 (3.5%) reflected minor CIP‐related shifts, particularly at higher concentrations upstream‐sourced. Prokaryotic communities exhibited weaker source site‐driven separation, with downstream‐sourced biofilms enriched in *Cyanobacteria* (e.g., *Leptolyngbya*) and upstream‐sourced communities associated with *Proteobacteria* and *Bacteroidetes*, consistent with univariate patterns in bacterial and phototrophic abundances under CIP exposure.

## 4 Discussion

### 4.1 Structural and Functional Differences Between Two Biofilm Communities Differing in Presumed WWTP Influence

Our results demonstrate that the source site (upstream vs. downstream of the WWTP) was associated with more variation in community structure and function, outweighing the effects of short‐term CIP exposure. Across biomass, pigment composition, FA profiles, taxonomic composition and diversity, upstream‐ and downstream‐sourced communities showed distinct patterns that are consistent with contrasting environmental histories. Under control conditions, upstream‐sourced biofilms exhibited higher chlorophyll concentrations and greater proportions of nutritionally important FAs, including PUFAs and Ω3 FAs. This pattern is consistent with a stronger contribution of diatoms and other phototrophs known to produce long‐chain Ω3 PUFAs, which are associated with high food quality for grazers (Huggins et al. 2004; Arts et al. 2009; Taipale et al. 2013; Yan et al. 2024). However, these upstream‐sourced communities accumulated less biomass and exhibited lower alpha diversity for eukaryotes, suggesting dominance of fewer, functionally specialised taxa. Such communities may be productive but potentially less buffered against disturbance, as reduced diversity can limit functional redundancy and stability (Allison and Martiny 2008; Feng et al. 2017; Baert et al. 2016). In contrast, downstream‐sourced biofilms were characterised by greater biomass and higher taxonomic diversity, with increasing relative abundance of *Chlorophyceae*, cyanobacteria and diverse bacterial taxa. Fast‐growing green algae, such as *Stigeoclonium* and *Uronema* are known for opportunistic resource use and high maximum growth rates (Reynolds 2006; Litchman and Klausmeier 2008), traits that may favour rapid biomass production under nutrient‐rich conditions. In addition, downstream‐sourced enrichment of *Cyanobacteria* and *Proteobacteria* is broadly consistent with patterns previously reported from other wastewater‐impacted systems (Tamminen et al. 2022; Chonova et al. 2019), where chronic exposure to nutrients, micropollutants and altered temperature regimes has been associated with shifts in periphytic communities. Continuous inflow of microbial propagules and contaminants from WWTPs is one plausible mechanism that could act as a persistent environmental filter (i.e., press disturbance) and select for taxa with metabolic flexibility, tolerance mechanisms and efficient growth strategies (Gillings et al. 2014; Marti et al. 2013; Silvester et al. 2025; Talat et al. 2025) although our study design does not allow this mechanism to be tested directly.

These observations are consistent with the hypothesis that long‐term press disturbance could establish a baseline community that shapes subsequent structural and functional responses (Bundschuh et al. 2017). However, because our design does not include replicated WWTP‐impacted or reference reaches, this remains an assumption rather than a demonstrated causal link, and taxonomic and trait differences between the two sites could equally reflect other unmeasured, spatially structured factors. The higher diversity and more even functional‐group distribution in downstream‐sourced biofilms are consistent with the portfolio concept (Schindler et al. 2015), whereby diversity may enhance stability by spreading risk across taxa with different response strategies (Cardinale 2011; Carles et al. 2021). However, because our study compares only a single source site pair, we present this as a plausible, testable explanation for the greater tolerance observed downstream rather than a demonstrated mechanism. Biofilms exposed to CIP were derived from upstream‐ and downstream‐sourced inocula after a 28‐day common‐garden incubation under identical laboratory conditions. The persistence of structural and functional differences following this incubation suggests that source site‐associated characteristics were retained despite community development under standardised conditions. Nevertheless, profiling the source communities prior to incubation would have helped quantify the extent of community change during laboratory growth and represents a valuable addition for future studies.

### 4.2 CIP Effects in the Context of Disturbance History

Although CIP exposure induced concentration‐dependent responses, its effects were consistently modulated by the source site. Upstream‐sourced biofilms exhibited stronger reductions in biomass, chlorophyll content, FA content and prokaryotic diversity. These changes were associated with declines in diatom‐dominated communities, particularly *Melosira*, taxa previously reported as sensitive to antibiotics and other pharmaceuticals (Robinson et al. 2005; Ebert et al. 2011). Given that diatoms are major contributors to highly unsaturated FA production and chlorophylls (Arts et al. 2009; Taipale et al. 2013), their reduction directly translated into lower pigment concentrations and diminished nutritional quality. Fluoroquinolones, such as CIP, interfere with DNA replication and may affect photosynthetic processes (Cleuvers 2003; Brain et al. 2009; Aristilde et al. 2010), providing a plausible mechanistic basis for the observed physiological and compositional shifts. In contrast, downstream‐sourced communities showed comparatively minor adverse structural and functional responses under the tested conditions. One apparent contradiction concerns downstream‐sourced biofilms, where CIP exposure reduced chlorophyll a, b and c concentrations (Figure 1) while green‐algal abundance increased (Figure 3a). This difference likely reflects the distinct nature of these measurements: qPCR‐based estimates specifically targeted green algae, whereas chlorophyll content integrates pigment contributions from the entire phototrophic community, including diatoms. As diatom abundance did not increase under CIP exposure (Figure 3c), shifts in phototrophic community composition may explain the decrease in bulk chlorophyll despite increased green‐algal abundance. Bacterial abundance remained stable or increased under CIP exposure, which may indicate enrichment of tolerant taxa, broadly consistent with patterns described for antibiotic‐impacted environments (Marti et al. 2013; Proia et al. 2016). The persistence of *Chlorophyceae* and *Proteobacteria*, taxa frequently associated with wastewater influence and stress tolerance (Reynolds 2006; Litchman and Klausmeier 2008), likely contributed to maintaining biomass and functional attributes. Such responses are consistent with the hypothesis of environmental filtering, whereby chronic prior exposure could select for assemblages with reduced sensitivity to subsequent chemical stress. However, because our study compares only a single source site pair, we present this as a plausible, testable explanation for the greater tolerance observed downstream rather than a demonstrated mechanism. Importantly, multivariate analyses confirmed that source site explained a substantially larger fraction of community variation than CIP treatment, particularly for eukaryotes. Even when CIP induced measurable changes, these occurred against a backdrop of persistent differences between the upstream‐ and downstream‐sourced communities. We hypothesise that these baseline differences may reflect prior environmental filtering, although this was not directly tested. Thus, CIP exposure can be viewed as a short‐term disturbance imposed on communities that already differed substantially, potentially due to long‐term wastewater influence, although other site‐specific factors cannot be excluded. The magnitude and direction of CIP effects were therefore contingent upon the pre‐existing state of the two source communities. Integrating metabarcoding, taxon abundance, pigment and FA data highlights how structural differences were associated with ecosystem‐relevant functional outcomes in this experiment. Upstream‐sourced communities, although initially characterised by high phototrophic quality, were more susceptible to antibiotic‐induced disruption, which could potentially reduce their nutritional value for higher trophic levels (Arts et al. 2009; Taipale et al. 2020). Downstream‐sourced biofilms, in contrast, maintained more stable biomass and FA composition despite compositional adjustments, indicating comparatively greater functional stability under the tested conditions and exposure duration. Functional stability in the face of compositional change has been observed in other wastewater‐influenced systems (Proia et al. 2016) and may point to a role for trait redundancy and adaptive capacity, though this study was not designed to test that mechanism directly.

Our finding that downstream‐sourced biofilms exhibited greater functional and structural tolerance is broadly consistent with the observations of Rosi et al. (2018), who reported that biofilms from a heavily urbanised stream maintained stable respiration under exposure to a pharmaceutical mixture including CIP, whereas biofilms from a less urbanised reach showed reduced respiration. However, Rosi et al. (2018) also observed the largest shifts in bacterial community composition at the most urbanised site, contrasting with the relatively stable prokaryotic diversity we observed for downstream‐sourced biofilms. Together, these findings suggest that functional and compositional tolerance to antibiotic exposure do not necessarily align and may depend on site‐specific environmental context and community history.

## 5 Conclusions

Our findings show that the source site of biofilms exerted a stronger and more persistent influence on biofilm structure and ecosystem functioning than short‐term CIP exposure under the conditions tested. We propose that continuous wastewater influence is one plausible driver of the baseline differences observed between the two source communities, although this hypothesis requires validation through replicated comparisons across multiple WWTP‐impacted and reference reaches. Although CIP altered biomass, pigments, FA and microbial composition, these responses were strongly context dependent: upstream‐sourced biofilms were generally more sensitive, whereas downstream‐sourced communities displayed greater tolerance. This pattern may reflect ecological preconditioning, higher diversity and greater functional redundancy, though these mechanisms were not directly tested. More broadly, our results suggest that disturbance history and baseline community structure can shape biofilm vulnerability to contamination, consistent with—though not a direct test of—the environmental filtering concept. By linking compositional, functional and biochemical responses, we highlight the potential importance of legacy effects and spatial heterogeneity in determining biofilm responses to contaminants and suggest that short‐term toxicity assays alone may underestimate community‐level impacts in systems exposed to long‐term press disturbances.

## Author Contributions

**Clara Benavent‐Celma:** conceptualization, methodology, formal analysis, investigation, validation, funding acquisition, visualization, project administration, resources, writing – original draft, writing – review and editing, supervision. **Sophie Oster:** investigation, writing – review and editing. **Sebastian Pietz:** investigation, writing – review and editing. **Sabine Filker:** formal analysis, investigation, methodology, validation, visualization, writing – original draft, writing – review and editing. **Mirco Bundschuh:** conceptualization, methodology, validation, supervision, project administration, writing – review and editing. **Timo Mühlhaus:** writing – review and editing. **Alexander Feckler:** conceptualization, methodology, investigation, validation, formal analysis, supervision, visualization, project administration, writing – original draft, writing – review and editing.

## Funding

This work was supported by the SULSA–Rheinland‐Pfalz Research Collaboration Funding 2023.

## Conflicts of Interest

The authors declare no conflicts of interest.

## Supporting information


**Table S1:** Composition of modified SAM‐S5 media (Borgmann 1996). Salts in bold letters display the modifications (Rybicki et al. 2012; Rybicki and Jungmann 2018).
**Table S2:** Sequences of ‘model amplicons’, their length (base pairs; plus end degradation buffer) and source of the sequence that was used for estimations of algal, bacterial, diatom and fungal operon copies. Primer binding sites are highlighted in bold and are underlined.
**Figure S1:** Posterior densities for the differences observed for biofilm (a) biomass, (b) chlorophyll *a* content, (c) chlorophyll *b* content, (d) chlorophyll *c*s content between the control and treatments containing 3 μg CIP L^−1^ (light blue), 31 μg CIP L^−1^ (dark blue), 310 μg CIP L^−1^ (light green). The Bayesian factor (BF) indicates the odds of the probabilities of differences being greater than zero and differences being smaller than zero (and vice versa as it is always expressed as > 1).
**Figure S2:** Posterior densities for the differences observed for biofilm (a) algal abundance, (b) bacterial abundance, (c) diatom abundance, (d) fungal abundance between the control and treatments containing 3 μg CIP L^−1^ (light blue), 31 μg CIP L^−1^ (dark blue), 310 μg CIP L^−1^ (light green). The Bayesian factor (BF) indicates the odds of the probabilities of differences being greater than zero and differences being smaller than zero (and vice versa as it is always expressed as > 1).
**Figure S3:** Comparison of alpha diversity of eukaryotic (a) and prokaryotic (b) biofilm communities between upstream and downstream‐sourced samples. Each facet shows a single metric (Shannon and Simpson index) with a boxplot displaying the median (central line), interquartile range (box) and whiskers extending to 1.5 × IQR, overlaid with jittered points for individual sample values.
**Table S3:** Mean ±95% credible interval for FA groups separated by origin and treatment.
**Table S4:** Outcome of the PERMANOVA on FA composition.
**Table S5:** Bayesian factors for FA groups.

## Data Availability

All data and code are publicly available on Zenodo (https://doi.org/10.5281/zenodo.20355858).

## References

[emi470408-bib-0001] Ahmed, M. N. , A. Porse , M. O. A. Sommer , N. Høiby , and O. Ciofu . 2018. “Evolution of Antibiotic Resistance in Biofilm and Planktonic *pseudomonas aeruginosa* Populations Exposed to Subinhibitory Levels of Ciprofloxacin.” Antimicrobial Agents and Chemotherapy 62, no. 8: 1–12. 10.1128/AAC.00320-18. PMC6105853 29760140

[emi470408-bib-0002] Al‐buriahi, A. K. , M. M. Al‐shaibani , R. M. S. R. Mohamed , A. A. Al‐Gheethi , A. Sharma , and N. Ismail . 2022. “Journal of Water Process Engineering Ciprofloxacin Removal From Non‐Clinical Environment: A Critical Review of Current Methods and Future Trend Prospects.” Journal of Water Process Engineering 47, no. March: 102725. 10.1016/j.jwpe.2022.102725.

[emi470408-bib-0003] Allison, S. D. , and J. B. H. Martiny . 2008. “Resistance, Resilience, and Redundancy in Microbial Communities.” Proceedings of the National Academy of Sciences of the United States of America 105, no. SUPPL. 1: 11512–11519. 10.1073/pnas.0801925105. 18695234 PMC2556421

[emi470408-bib-0004] Aristilde, L. , A. Melis , and G. Sposito . 2010. “Inhibition of Photosynthesis by a Fluoroquinolone Antibiotic.” Environmental Science and Technology 44, no. 4: 1444–1450. 10.1021/es902665n. 20070075

[emi470408-bib-0005] Arts, M. , M. Brett , and M. Kainz . 2009. Lipids in Aquatic Ecosystems. Springer Nature.

[emi470408-bib-0006] Baert, J. M. , F. De Laender , K. Sabbe , and C. Janssen . 2016. “Biodiversity Increases Functional and Compositional Resistance, but Decreases Resilience in Phytoplankton Communities.” Ecology 97, no. 12: 3433–3440. 10.1002/ecy.1601. 27912020

[emi470408-bib-0007] Baker, G. C. , J. J. Smith , and D. A. Cowan . 2003. “Review and Re‐Analysis of Domain‐Specific 16S Primers.” Journal of Microbiological Methods 55: 541–555. 10.1016/j.mimet.2003.08.009. 14607398

[emi470408-bib-0008] Battin, T. J. , K. Besemer , M. M. Bengtsson , A. M. Romani , and A. I. Packmann . 2016. “The Ecology and Biogeochemistry of Stream Biofilms.” Nature Reviews Microbiology 14, no. April: 251–263. 26972916 10.1038/nrmicro.2016.15

[emi470408-bib-0009] Benavent‐Celma, C. , S. Oster , S. Pietz , et al. 2026. Biofilm Source Shapes the Tolerance to Short‐Term Ciprofloxacin Exposure. Zenodo. 10.5281/zenodo.20355858. PMC13526528 42669567

[emi470408-bib-0010] Bernhardt, E. S. , E. J. Rosi , and M. O. Gessner . 2017. Synthetic Chemicals as Agents of Global Change. WHO. 10.1002/fee.1450.

[emi470408-bib-0011] Bokulich, N. A. , S. Subramanian , J. J. Faith , et al. 2013. “Quality‐Filtering Vastly Improves Diversity Estimates From Illumina Amplicon Sequencing.” 10, no. 1: 57–60. 10.1038/NMETH.2276. PMC3531572 23202435

[emi470408-bib-0012] Booth, A. , D. S. Aga , and A. L. Wester . 2020. “Retrospective Analysis of the Global Antibiotic Residues That Exceed the Predicted no Effect Concentration for Antimicrobial Resistance in Various Environmental Matrices.” Environment International 141, no. April: 105796. 10.1016/j.envint.2020.105796. 32422499

[emi470408-bib-0013] Borgmann, U. 1996. “Systematic Analysis of Aqueous Ion Requirements of *Hyalella azteca* : A Standard Artificial Medium Including the Essential Bromide Ion.” Archives of Environmental Contamination and Toxicology 363: 356–363.

[emi470408-bib-0014] Brain, R. A. , K. R. Solomon , and B. W. Brooks . 2009. “Targets , Effects and Risks in Aquatic Plants Exposed to Veterinary Antibiotics.” In Veterinary Pharmaceuticals in the Environment (ACS Symposium Series), edited by K. L. Henderson and J. R. Coats , Vol. 1018, 169–189. American Chemical Society.

[emi470408-bib-0015] Bundschuh, M. , R. Schulz , R. B. Schäfer , C. R. Allen , and D. G. Angeler . 2017. “Resilience in Ecotoxicology: Towards a Multiple Equilibrium Concept.” Environmental Toxicology and Chemistry 36: 2574–2580. 10.1002/etc.3839. 28493505

[emi470408-bib-0016] Bürkner, P.‐C. 2017. “brms: An R Package for Bayesian Multilevel Models.” Journal of Statistical Software 80, no. 1: 1–28. 10.18637/jss.v080.i01.

[emi470408-bib-0017] Cadotte, M. W. , and C. M. Tucker . 2017. “Should Environmental Filtering Be Abandoned ?” Trends in Ecology & Evolution 32, no. 6: 429–437. 10.1016/j.tree.2017.03.004. 28363350

[emi470408-bib-0018] Callahan, B. J. , P. J. McMurdie , M. J. Rosen , A. W. Han , A. J. A. Johnson , and S. P. Holmes . 2016. “DADA2 : High‐Resolution Sample Inference From Illumina Amplicon Data.” Nature Methods 13, no. 7: 581–583. 10.1038/nmeth.3869. 27214047 PMC4927377

[emi470408-bib-0019] Cardinale, B. J. 2011. “Biodiversity Improves Water Quality Through Niche Partitioning.” Nature 3: 2–7. 10.1038/nature09904. 21475199

[emi470408-bib-0020] Carles, L. , S. Wullschleger , A. Joss , et al. 2021. “Impact of Wastewater on the Microbial Diversity of Periphyton and Its Tolerance to Micropollutants in an Engineered Flow‐Through Channel System.” Water Research 203: 117486. 10.1016/j.watres.2021.117486. 34412020

[emi470408-bib-0096] Castellani, A. , and A. J. Chalmers . 1919. Manual of Tropical Medicine. 3rd ed., 941. William Wood and Company.

[emi470408-bib-0021] Chonova, T. , R. Kurmayer , F. Rimet , et al. 2019. “Benthic Diatom Communities in an Alpine River Impacted by Waste Water Treatment Effluents as Revealed Using DNA Metabarcoding.” Frontiers in Microbiology 10, no. April: 1–17. 10.3389/fmicb.2019.00653. 31024473 PMC6465766

[emi470408-bib-0022] Clarke, K. R. 1993. “Non‐Parametric Multivariate Analyses of Changes in Community Structure.” Australian Journal of Ecology 18, no. 1: 117–143. 10.1111/j.1442-9993.1993.tb00438.x.

[emi470408-bib-0023] Cleuvers, M. 2003. “Aquatic Ecotoxicity of Pharmaceuticals Including the Assessment of Combination Effects.” Toxicology Letters 142: 185–194. 10.1016/S0378-4274(03)00068-7. 12691712

[emi470408-bib-0024] Ebert, I. , J. Bachmann , U. Kühnen , et al. 2011. “Toxicity of the Fluoroquinolone Antibiotics Enrofloxacin and Ciprofloxacin to Photoautotrophic Aquatic Organisms.” Environmental Toxicology and Chemistry 30, no. 12: 278. 10.1002/etc.678 21919043

[emi470408-bib-0093] Edgar, R. C. 2016. “SINTAX: A Simple Non‐Bayesian Taxonomy Classifier for 16S and ITS Sequences.” bioRxiv, 074161. 10.1101/074161.

[emi470408-bib-0025] Elwood, H. J. , J. Gary , and M. L. Sogin . 1985. “The Small‐Subunit Ribosomal RNA Gene Sequences From the Hypotrichous Ciliates *Oxytriclha nova* and *Stylonychia pustulata* .” Molecular Biology and Evolution 2, no. 5: 399–410. 3939705 10.1093/oxfordjournals.molbev.a040362

[emi470408-bib-0026] Ewing, B. , and P. Green . 1998. “Base‐Calling of Automated Sequencer Traces Using Phred. II. Error Probabilities.” Genome Research 206: 186–194. 9521922

[emi470408-bib-0027] Feckler, A. , P. Baudy‐Groh , L. Friedrichs , et al. 2023. “Diatoms Reduce Decomposition of and Fungal Abundance on Less Recalcitrant Leaf Litter via Negative Priming.” Microbial Ecology 86: 2674. 37505287 10.1007/s00248-023-02268-w PMC10640500

[emi470408-bib-0028] Feckler, A. , J. Rakovic , M. Kahlert , R. Tröger , and M. Bundschuh . 2018. “Blinded by the Light: Increased Chlorophyll Fluorescence of Herbicide‐Exposed Periphyton Masks Unfavorable Structural Responses During Exposure and Recovery.” Aquatic Toxicology 203, no. August: 187–193. 10.1016/j.aquatox.2018.08.015. 30153560

[emi470408-bib-0029] Feckler, A. , R. Schulz , and R. B. Schäfer . 2025. Meta Analysis on the Effects of Chemical Stressors on Freshwater Ecosystem Functions.

[emi470408-bib-0030] Feng, K. , Z. Zhang , W. Cai , et al. 2017. “Biodiversity and Species Competition Regulate the Resilience of Microbial Biofilm Community.” Molecular Ecology 26, no. 21: 6170–6182. 10.1111/mec.14356. 28926148

[emi470408-bib-0031] Fick, J. , H. Söderström , R. H. Lindberg , C. Phan , M. Tysklind , and D. G. J. Larsson . 2009. “Contamination of Surface, Ground, and Drinking Water From Pharmaceutical Production.” Environmental Toxicology and Chemistry 28, no. 12: 2522–2527. 10.1897/09-073.1. 19449981

[emi470408-bib-0032] Fierer, N. , J. A. Jackson , R. Vilgalys , and R. B. Jackson . 2005. “Assessment of Soil Microbial Community Structure by Use of Taxon‐Specific Quantitative PCR Assays.” Applied and Environmental Microbiology 71, no. 7: 4117–4120. 10.1128/AEM.71.7.4117-4120.2005. 16000830 PMC1169028

[emi470408-bib-0033] Fink, P. 2013. “Invasion of Quality: High Amounts of Essential Fatty Acids in the Invasive Ponto‐Caspian Mysid *Limnomysis benedeni* .” Journal of Plankton Research 35: 907–913. 10.1093/plankt/fbt029.

[emi470408-bib-0034] Gallagher, M. T. , and A. J. Reisinger . 2020. “Effects of Ciprofloxacin on Metabolic Activity and Algal Biomass of Urban Stream Biofilms.” Science of the Total Environment 706: 135728. 10.1016/j.scitotenv.2019.135728. 31940730

[emi470408-bib-0035] Gillings, M. R. , W. H. Gaze , A. Pruden , K. Smalla , J. M. Tiedje , and Y. G. Zhu . 2014. “Using the Class 1 Integron‐Integrase Gene as a Proxy for Anthropogenic Pollution.” ISME Journal 9, no. 6: 1269–1279. 10.1038/ismej.2014.226. 25500508 PMC4438328

[emi470408-bib-0036] Godhe, A. , M. E. Asplund , and K. Ha . 2008. “Quantification of Diatom and Dinoflagellate Biomasses in Coastal Marine Seawater Samples by Real‐Time PCR.” Applied and Environmental Microbiology 74, no. 23: 7174–7182. 10.1128/AEM.01298-08. 18849462 PMC2592920

[emi470408-bib-0037] González‐Pleiter, M. , S. Gonzalo , I. Rodea‐Palomares , et al. 2013. “Toxicity of Five Antibiotics and Their Mixtures Towards Photosynthetic Aquatic Organisms : Implications for Environmental Risk Assessment.” Water Research 47, no. 6: 2. 10.1016/j.watres.2013.01.020. 23399078

[emi470408-bib-0038] Grenni, P. , V. Ancona , and A. Barra . 2018. “Ecological Effects of Antibiotics on Natural Ecosystems: A Review.” Microchemical Journal 136: 25–39. 10.1016/j.microc.2017.02.006.

[emi470408-bib-0039] Guillou, L. , D. Bachar , S. Audic , et al. 2013. “The Protist Ribosomal Reference Database (PR 2): A Catalog of Unicellular Eukaryote Small Sub‐Unit rRNA Sequences With Curated Taxonomy.” Nucleic Acids Research 41: 597–604. 10.1093/nar/gks1160. PMC3531120 23193267

[emi470408-bib-0040] Hill, W. R. , and S. M. Dimick . 2002. “Effects of Riparian Leaf Dynamics on Periphyton Photosynthesis and Light Utilisation Efficiency.” Freshwater Biology 47: 1245–1256. 10.1046/j.1365-2427.2002.00837.x.

[emi470408-bib-0041] Huggins, K. I. M. , J. Frenette , and M. T. Arts . 2004. “Nutritional Quality of Biofilms With Respect to Light Regime in Lake Saint‐Pierre (Quebec Canada).” Freshwater Biology 49: 945–959.

[emi470408-bib-0042] Jeffrey, S. , and G. Humphrey . 1975. “New Spectrophotometric Method for Determining Chlorophyll a, b, Cl and c2 in Algae, Phytoplankton, and Higher Plants.” Biochemie Und Physiologie der Pflanzen 167: 191–194.

[emi470408-bib-0043] Konschak, M. , J. P. Zubrod , P. Baudy , et al. 2020. “The Importance of Diet‐Related Effects of the Antibiotic Ciprofloxacin on the Leaf‐Shredding Invertebrate Gammarus Fossarum (Crustacea; Amphipoda).” Aquatic Toxicology 222, no. August 2019: 105461. 10.1016/j.aquatox.2020.105461. 32171118

[emi470408-bib-0044] Kümmerer, K. 2009. “Antibiotics in the Aquatic Environment: A Review—Part I.” Chemosphere 75, no. 4: 417–434. 10.1016/j.chemosphere.2008.11.086. 19185900

[emi470408-bib-0045] Lamprecht, O. , B. Wagner , N. Derlon , and A. Tlili . 2022. “Synthetic Periphyton as a Model System to Understand Species Dynamics in Complex Microbial Freshwater Communities.” npj Biofilms and Microbiomes 8, no. 1: 61. 10.1038/s41522-022-00322-y. 35869094 PMC9307524

[emi470408-bib-0046] Lauridsen, T. L. , L. Schlüter , and L. S. Johansson . 2011. “Determining Algal Assemblages in Oligotrophic Lakes and Streams: Comparing Information From Newly Developed Pigment/Chlorophyll a Ratios With Direct Microscopy.” Freshwater Biology 56, no. 8: 1638–1651. 10.1111/j.1365-2427.2011.02588.x.

[emi470408-bib-0048] Litchman, E. , and C. A. Klausmeier . 2008. “Trait‐Based Community Ecology of Phytoplankton.” Annual Review of Ecology, Evolution, and Systematics 39: 615–639. 10.1146/annurev.ecolsys.39.110707.173549.

[emi470408-bib-0049] Łukaszewicz, P. , J. Maszkowska , E. Mulkiewicz , J. Kumirska , and P. Stepnowski 2017. “Impact of Veterinary Pharmaceuticals on the Agricultural Environment: A Re‐Inspection.” Reviews of Environmental Contamination and Toxicology 243: 89–148. 10.1007/398_2016_16. 28005213

[emi470408-bib-0050] Manerkar, M. A. , S. Seena , and F. Bärlocher . 2014. “Q‐RT‐PCR for Assessing Archaea , Bacteria, and Fungi During Leaf Q‐RT‐PCR for Assessing Archaea , Bacteria , and Fungi During Leaf Decomposition in a Stream.” Microbial Ecology 56, no. 3: 467–473. 10.1007/s00248-008-9365-z. 18264658

[emi470408-bib-0051] Marizzi del Olmo, A. , J. C. López‐Doval , M. Hidalgo , et al. 2025. “Holistic Assessment of Chemical and Biological Pollutants in a Mediterranean Wastewater Effluent‐Dominated Stream: Interactions and Ecological Impacts.” Environmental Pollution 370: 125833. 10.1016/j.envpol.2025.125833. 39952585

[emi470408-bib-0052] Marti, E. , J. Jofre , and J. L. Balcazar . 2013. “Prevalence of Antibiotic Resistance Genes and Bacterial Community Composition in a River Influenced by a Wastewater Treatment Plant.” PLoS One 8, no. 10: 1–8. 10.1371/journal.pone.0078906. PMC3808343 24205347

[emi470408-bib-0053] Martin, M. 2011. “Cutadapt Removes Adapter Sequences From High‐Throughput Sequencing Reads.” EMBnet.Journal 17, no. 1: 10–12. 10.14806/ej.17.1.200.

[emi470408-bib-0054] Meyer, F. E. , L. S. Shuey , S. Naidoo , et al. 2016. “Dual RNA‐Sequencing of *Eucalyptus nitens* During Phytophthora Cinnamomi Challenge Reveals Pathogen and Host Factors Influencing Compatibility.” Frontiers in Plant Science 7: 1–15. 10.3389/fpls.2016.00191. 26973660 PMC4773608

[emi470408-bib-0055] Miao, Y. , N. W. Johnson , P. B. Gedalanga , D. Adamson , C. Newell , and S. Mahendra . 2019. “Response and Recovery of Microbial Communities Subjected to Oxidative and Biological Treatments of 1,4‐Dioxane and Co‐Contaminants.” Water Research 149: 74–85. 10.1016/j.watres.2018.10.070. 30419469

[emi470408-bib-0056] Nie, X. , J. Gu , J. Lu , W. Pan , and Y. Yang . 2009. “Effects of Norfloxacin and Butylated Hydroxyanisole on the Freshwater Microalga *Scenedesmus obliquus* .” Ecotoxicology 18, no. 6: 677–684. 10.1007/s10646-009-0334-1. 19495962

[emi470408-bib-0092] Oksanen, J. , G. L. Simpson , F. G. Blanchet , et al. 2022. “Vegan: Community Ecology Package.” R Package Version 2.6–2.

[emi470408-bib-0057] Oster, S. 2025. “Functional Stability Despite Structural Changes in Freshwater Biofilm Communities Exposed to an Antibiotic and an Herbicide ‐ The Role of Nutrient Conditions.” FEMS Microbiology Ecology 101: fiaf094. 10.1093/femsec/fiaf094. 40990949 PMC12481197

[emi470408-bib-0058] Pietz, S. , S. Kolbenschlag , N. Röder , et al. 2023. “Subsidy Quality Affects Common Riparian Web‐Building Spiders: Consequences of Aquatic Contamination and Food Resource.” Environmental Toxicology and Chemistry 42, no. 6: 1346–1358. 10.1002/etc.5614. 36946335

[emi470408-bib-0059] Porsbring, T. , Å. Arrhenius , T. Backhaus , et al. 2007. “The SWIFT Periphyton Test for High‐Capacity Assessments of Toxicant Effects on Microalgal Community Development.” Journal of Experimental Marine Biology and Ecology 349: 299–312. 10.1016/j.jembe.2007.05.020.

[emi470408-bib-0060] Proia, L. , D. von Schiller , A. Sànchez‐Melsió , et al. 2016. “Occurrence and Persistence of Antibiotic Resistance Genes in River Biofilms After Wastewater Inputs in Small Rivers.” Environmental Pollution 210: 121–128. 10.1016/j.envpol.2015.11.035. 26708766

[emi470408-bib-0061] Quast, C. , E. Pruesse , P. Yilmaz , et al. 2013. “The SILVA Ribosomal RNA Gene Database Project : Improved Data Processing and Web‐Based Tools.” Nucleic Acids Research 41: 590–596. 10.1093/nar/gks1219. PMC3531112 23193283

[emi470408-bib-0094] R Core Team . 2025. R: A Language and Environment for Statistical Computing. R Foundation for Statistical Computing. https://www.R‐project.org/.

[emi470408-bib-0062] Reynolds, C. S. 2006. The Ecology of Phytoplankton. Cambridge University Press.

[emi470408-bib-0063] Richmond, E. K. , E. J. Rosi , D. M. Walters , et al. 2018. “A Diverse Suite of Pharmaceuticals Contaminates Stream and Riparian Food Webs.” Nature Communications 9, no. 1: 1–9. 10.1038/s41467-018-06822-w. PMC6219508 30401828

[emi470408-bib-0064] Rivera, S. F. , V. Vasselon , A. Bouchez , and F. Rimet . 2023. “eDNA Metabarcoding From Aquatic Biofilms Allows Studying Spatial and Temporal Fluctuations of Fish Communities From Lake Geneva.” Environmental DNA 5, no. 3: 570–581. 10.1002/edn3.413.

[emi470408-bib-0065] Robinson, A. A. , J. B. Belden , and M. J. Lydy 2005. “Toxicity of Fluoroquinolone Antibiotics to Aquatic Organisms. Environ.” Toxicological Chemistry 24, no. 2: 423. 10.1897/04-210r.1 15720004

[emi470408-bib-0066] Rodriguez‐mozaz, S. , S. Chamorro , E. Marti , et al. 2014. “Occurrence of Antibiotics and Antibiotic Resistance Genes in Hospital and Urban Wastewaters and Their Impact on the Receiving River.” Water Research 69: 234–242. 10.1016/j.watres.2014.11.021. 25482914

[emi470408-bib-0067] Rognes, T. , T. Flouri , B. Nichols , et al. 2016. “VSEARCH: A Versatile Open Source Tool for Metagenomics.” PeerJ 4: e2584. 10.7717/peerj.2584. 27781170 PMC5075697

[emi470408-bib-0068] Rosi, E. J. , H. A. Bechtold , D. Snow , M. Rojas , A. J. Reisinger , and J. J. Kelly . 2018. “Urban Stream Microbial Communities Show Resistance to Pharmaceutical Exposure.” Ecosphere 9, no. 1: e02041. 10.1002/ecs2.2041.

[emi470408-bib-0069] Rybicki, M. , and D. Jungmann . 2018. “Direct and Indirect Effects of Pesticides on a Benthic Grazer During Its Life Cycle.” Environmental Sciences Europe 30: 35. 10.1186/s12302-018-0165-x. 30294514 PMC6153858

[emi470408-bib-0070] Rybicki, M. , C. Winkelmann , C. Hellmann , P. Bartels , and D. Jungmann . 2012. “Herbicide Indirectly Reduces Physiological Condition of a Benthic Grazer.” Aquatic Biology 17: 153–166.

[emi470408-bib-0071] Schindler, D. E. , J. B. Armstrong , and T. E. Reed . 2015. “The Portfolio Concept in Ecology and Evolution.” Frontiers in Ecology and the Environment 13, no. 5: 257–263. 10.1890/140275.

[emi470408-bib-0072] Silvester, R. , W. B. Perry , G. Webser , et al. 2025. “Metagenomics Unveils the Role of Hospitals and Wastewater Treatment Plants on the Environmental Burden of Antibiotic Resistance Genes and Opportunistic Pathogens.” Science of the Total Environment 961: 178403. 39798461 10.1016/j.scitotenv.2025.178403

[emi470408-bib-0073] Simon, N. , L. Campbell , E. Örnolfsdottir , et al. 2000. “Oligonucleotide Probes for the Identification of Three Algal Groups by Dot Blot and Fluorescent Whole‐Cell Hybridization.” Journal of Eukaryotic Microbiology 47, no. 1: 76–84. 10.1111/j.1550-7408.2000.tb00014.x. 10651300

[emi470408-bib-0095] Stan Development Team . 2024. Stan User's Guide, VERSION. https://mc‐stan.org.

[emi470408-bib-0074] Steinman, A. D. , G. A. Lamberti , and P. R. Leavitt . 2006. “Biomass and Pigments of Benthic Algae.” In Methods in Stream Ecology, edited by F. R. Hauer and G. A. Lamberti , 2nd ed., 357–379. Academic Press.

[emi470408-bib-0075] Stoeck, T. , D. Bass , M. Nebel , et al. 2010. “Multiple Marker Parallel Tag Environmental DNA Sequencing Reveals a Highly Complex Eukaryotic Community in Marine Anoxic Water.” Molecular Ecology 19: 21–31. 10.1111/j.1365-294X.2009.04480.x. 20331767

[emi470408-bib-0091] Sturm, M. T. , E. Myers , D. Schober , C. Thege , A. Korzin , and K. Schuhen . 2022. “Adaptable Process Design as a Key for Sustainability Upgrades in Wastewater Treatment: Comparative Study on the Removal of Micropollutants by Advanced Oxidation and Granular Activated Carbon Processing at a German Municipal Wastewater Treatment Plant.” Sustainability 14, no. 18: 11605. 10.3390/su141811605.

[emi470408-bib-0076] Taipale, S. , E. Peltomaa , and P. Salmi . 2020. “Variation in ω −3 and ω −6 Polyunsaturated Fatty Acids Produced by Di Ff Erent Phytoplankton Taxa at Early and Late Growth Phase.” Biomolecules 10, no. 4: 559. 32268552 10.3390/biom10040559 PMC7226532

[emi470408-bib-0077] Taipale, S. , U. Strandberg , E. Peltomaa , et al. 2013. “Fatty Acid Composition as Biomarkers of Freshwater Microalgae: Analysis of 37 Strains of Microalgae in 22 Genera and in Seven Classes.” Aquatic Microbial Ecology 71, no. 1: 165–178. 10.3354/ame01671.

[emi470408-bib-0078] Talat, A. , Y. Bashir , N. Khalil , et al. 2025. ‘Antimicrobial Resistance Transmission in the Environmental Settings Through Traditional and UV‐Enabled Advanced Wastewater Treatment Plants: A Metagenomic Insight’. 10.1186/s40793-024-00658-2 PMC11884044 40050994

[emi470408-bib-0079] Tamminen, M. , J. Spaak , A. Tlili , R. Eggen , C. Stamm , and K. Räsänen . 2022. “Wastewater Constituents Impact Biofilm Microbial Community in Receiving Streams.” Science of the Total Environment 807: 151080. 10.1016/j.scitotenv.2021.151080. 34678363

[emi470408-bib-0080] Tlili, A. , J. Hollender , C. Kienle , and R. Behra . 2017. “Micropollutant‐Induced Tolerance of in Situ Periphyton : Establishing Causality in Wastewater‐Impacted Streams.” Water Research 111: 185–194. 10.1016/j.watres.2017.01.016. 28088715

[emi470408-bib-0081] Uruén, C. , G. Chopo‐Escuin , J. Tommassen , et al. 2021. “Biofilms as Promoters of Bacterial Antibiotic Resistance and Tolerance.” Antibiotics 10, no. 1: 1–36. 10.3390/antibiotics10010003. PMC7822488 33374551

[emi470408-bib-0082] Vannote, R. L. , G. W. Minshall , K. W. Cummins , et al. 1980. “The River Continuum Concept.” Canadian Journal of Fisheries and Aquatic Sciences 37, no. 1: 130–137.

[emi470408-bib-0083] Vinebrooke, R. D. , K. L. Cottingham , M. S. Norberg , S. I. Dodson , S. C. Maberly , and U. Sommer . 2004. “Impacts of Multiple Stressors on Biodiversity and Ecosystem Functioning: The Role of Species Co‐Tolerance.” Oikos 104, no. 3: 451–457. 10.1111/j.0030-1299.2004.13255.x.

[emi470408-bib-0084] Visek, W. J. 1978. “The Mode of Growth Promotion by Antibiotics.” Journal of Animal Science 46: 1447–1469.

[emi470408-bib-0085] Walters, W. , E. R. Hyde , D. Berg‐Lyons , et al. 2015. “Transcribed Spacer Marker Gene Primers for Microbial Community Surveys.” mSystems 1, no. 1: e00009‐15. 10.1128/mSystems.00009-15. 27822518 PMC5069754

[emi470408-bib-0086] Wangberg, B. H. 1988. “Induced Community Tolerance in Marine Periphyton Established Under Arsenate Stress.” Canadian Journal of Fisheries and Aquatic Sciences 45: 1816–1819.

[emi470408-bib-0087] Wetzel, R. G. 1983. Periphyton of Freshwater Ecosystems: Proceedings of the First International Workshop on Periphyton of Freshwater Ecosystems, Held in Växjö, Sweden.

[emi470408-bib-0088] White, T. J. , T. D. Bruns , S. B. Lee , and J. W. Taylor . 1990. ‘Amplification and Direct Sequencing of Fungal Ribosomal RNA Genes for Phylogenetics’.

[emi470408-bib-0089] Yan, K. , F. Guo , M. J. Kainz , et al. 2024. “The Importance of Omega‐3 Polyunsaturated Fatty Acids as High‐Quality Food in Freshwater Ecosystems With Implications of Global Change.” 86: 200–218. 10.1111/brv.13017. 37724488

[emi470408-bib-0097] Yeoh, Y. K. , C. Paungfoo‐Lonhienne , P. G. Dennis , et al. 2016. “The Core Root Microbiome of Sugarcanes Cultivated Under Varying Nitrogen Fertilizer Application.” Environmental Microbiology 18, no. 5: 1338–1351. 10.1111/1462-2920.12925. 26032777

[emi470408-bib-0090] Yeoh, Y. K. , Y. Sekiguchi , D. H. Parks , and P. Hugenholtz . 2016. “Comparative Genomics of Candidate Phylum TM6 Suggests That Parasitism Is Widespread and Ancestral in This Lineage.” Molecular Biology and Evolution 33, no. 4: 915–927. 10.1093/molbev/msv281. 26615204 PMC4776705

