# Supplementary material for: Biofilm Source Shapes the Tolerance to Short‐Term Ciprofloxacin Exposure
Source: Environ Microbiol Rep. 2026 Aug 30;18(5):e70408. doi: 10.1111/1758-2229.70408 (PMC13526528; doi:10.1111/1758-2229.70408)
Supplement: Supplementary file 1 [file EMI4-18-e70408-s001.docx]

**Biofilm source shapes the tolerance to short-term ciprofloxacin exposure**

Clara Benavent-Celma^1^ (ORCID: 0000-0003-2389-1769), Sophie Oster^2^ (ORCID: 0000-0003-4639-7384), Sebastian Pietz^2^ (ORCID: 0000-0002-2224-073X), Sabine Filker^3^ (ORCID: 0000-0002-4544-8277), Mirco Bundschuh^2^ (ORCID: 0000-0003-4876-220X), Timo Mühlhaus^3^ (ORCID: 0000-0003-3925-6778), Alexander Feckler^2^ (ORCID: 0000-0001-7836-4582)

^1^The James Hutton Institute, Craigiebuckler, Aberdeen, Scotland

^2^RPTU University Kaiserslautern-Landau, Department of Natural and Environmental Sciences, iES Landau, Institute for Environmental Sciences, Landau, Germany

^3^RPTU University Kaiserslautern-Landau, Department of Biology, Kaiserslautern, Germany

Contact details for the corresponding author: clara.benaventcelma@hutton.ac.uk

Table S1: Composition of modified SAM-S5 media (Borgmann, 1996). Salts in bold letters display the modifications (Rybicki et al., 2012; Rybicki & Jungmann, 2018).

| Salt | Quantity (mg L^-1^) |
| --- | --- |
| NaHCO_3_ | 85.55 |
| KCl | 3.8 |
| NaBr | 1.03 |
| MgSO_4_x7H_2_O | 61.5 |
| CaCl_2_x2H_2_O | 147 |
| **NaNO**_3_ | **9** |
| **Na_2_SiO_3_x5H_2_**O | **5** |
| **Na_2_HPO_4_** | **0.03** |

Table S2: Sequences of ‘model amplicons’, their length (base pairs; plus end degradation buffer), and source of the sequence that was used for estimations of algal, bacterial, diatom, and fungal operon copies. Primer binding sites are highlighted in bold and are underlined.

| Organism group | Sequence | Length (bp) | Source |
| --- | --- | --- | --- |
| Algae | GTGCCAGCAG**CCGCGGTAATTCCAGCTC**CAATAGCGTATATTTAAGTTGTTGCAGTTAAAAAGCTCGTAGTTGGATTTCGGGTGGGTTTCAGCGGTCCGCCTATGGTGAGTACTGCTGTGGCCTATCTTACTGTCGGGGACCTGCTTCTGGGCTTCATTGTCCGGGACAGGGATTCGGCATGGTTACTTTGAGTAAATTAGAGTGTTCAAAGCAGGCTTACGCCGTGAATACTTTAGCATGGAATAACATGATAGGACTCTGCCCTATTCTGTTGGCCTGTAGGAGTGGAGTAATGATTAAGAGGAACAGTCGGGGGCATTCGTATTTCATTGTCAGAGGTGAAATTCTTGGATTTATGAAAGACGAACTACTGCGAAAGCATTTGCCAAGGATGTTTTCATTAATCAAGAAC**GAAAGTTGGGGGCTCGAAG**ACGATTAGAT | 442 | In-house culture |
| Bacteria | AAATTGA**AGAGTTTGATCATGGCTCAG**ATTGAACGCTGGCGGCAGGCCTAACACATGCAAGTCGAACGGTAACAGGAAGAAGCTTGCTTCTTTGCTGACGAGTGGCGGACGGGTGAGTAATGTCTGGGAAACTGCCTGATGGGCCTCTTGCCATCGGATGTGCCCAGATGGGATTAGCTAGTAGGTGGGGTAACGGCTCACCTAGGCGACGATCCCTAGCTGGTCTGAGAGGATGACCAGCCACACTGGAACTGAGACACGGTCCAGACTCCTACGGGACGCAGCAGTGGGGAATATTGCACAATGGGCGCAAGCCTGATGCAGCCATGCCGCGTGTATGAAGAAGGCCTTCGGGTTGTAAAGTACTTTCAGCGGGGAGGAAGGGAGTAAAGTTAATACCTTTGCTCATTGACGTTACCCGCAGAAGAAGCACCGGCTAACTCC**GTGCCAGCAGCCGCGGTAA**CTCCGTG | 470 | Baker at al. (2003) |
| Diatoms | GGGGCAATAA**CAGGTCTGTGATGCCCTT**AGATGTTCTGGGCCGCACGCGCGCTACACTGATGCATTCAACGAGTTTTTCCTTGGCCGAGAGGCCTGGGCAATCTTTTGAACGTGCATCGTGATAGGGATAGATTATTGCAATTATTAATCTTGAACGAGGAATTCCTAGTAAACG**CAGATCATCAATCTGCATTG**ATTACGTCCC | 205 | In-house culture |
| Fungi | CTTGGCTCTG**GCATCGATGAAGAACGCAGC**GAAATGCGATAAGTAATGTGAATTGCAGAATTCAGTGAATCATCGAATCTTTGAACGCACATTGCGCCCCTTGGTATTCCGAGGGGCATGCCTATTCGAGCGTCATTATCACCCCTCAAGCTCAGCTTGGTGTTGAGGCCTGCTGTCAAGGCAGCCTCTAAAAGCAGTGGCAGTGCTGTCAGGCTCTAAGCGTAGTAATCTCTCTCGCTACAGACACCTGATGGACACTCGCCAGAACCCCCCATCTTTTAATGATTGACCTCGGATTAGGTAGGGATACCCGCTGAACTTAA**GCATATCAATAAGCGGAGGA**AAAGAAACCA | 353 | In-house culture |

Figure S1: Posterior densities for the differences observed for biofilm (a) biomass, (b) chlorophyll *a* content, (c) chlorophyll *b* content, (d) chlorophyll *c*s content between the control and treatments containing 3 µg CIP L-^1^ (light blue), 31 µg CIP L-^1^ (dark blue), 310 µg CIP L-^1^ (light green). The Bayesian factor (BF) indicates the odds of the probabilities of differences being greater than zero and differences being smaller than zero (and vice versa as it is always expressed as >1).

Figure S2: Posterior densities for the differences observed for biofilm (a) algal abundance, (b) bacterial abundance, (c) diatom abundance, (d) fungal abundance between the control and treatments containing 3 µg CIP L-^1^ (light blue), 31 µg CIP L-^1^ (dark blue), 310 µg CIP L-^1^ (light green). The Bayesian factor (BF) indicates the odds of the probabilities of differences being greater than zero and differences being smaller than zero (and vice versa as it is always expressed as >1).

Figure S3: Comparison of alpha diversity of eukaryotic (a) and prokaryotic (b) biofilm communities between upstream and downstream-sourced samples. Each facet shows a single metric (Shannon and Simpson index) with a boxplot displaying the median (central line), interquartile range (box), and whiskers extending to 1.5×IQR, overlaid with jittered points for individual sample values.

Table S3: Mean ± 95% credible interval for FA groups separated by origin and treatment

| Origin | Ciprofloxacin treatment | SAFA | MUFA | PUFA | Omega 3 |
| --- | --- | --- | --- | --- | --- |
| Upstream-sourced | 0 | 98.05 ± 36.06 | 141.42 ± 40.96 | 56.21 ± 22.61 | 43.34 ± 17.23 |
|  | 3 | 83.27 ± 14.47 | 120.53 ± 29.06 | 43.78 ± 6.21 | 33.52 ± 5.01 |
|  | 31 | 79.94 ± 24.59 | 115.49 ± 34.05 | 47.71 ± 15.92 | 35.91 ± 11.55 |
|  | 310 | 63.26 ± 5.92 | 82 ± 3.03 | 36.29 ± 5.17 | 27.3 ± 4.01 |
| Downstream-sourced | 0 | 34.26 ± 3.55 | 33.55 ± 5.73 | 29.17 ± 3.04 | 21.28 ± 2.39 |
|  | 3 | 30.13 ± 7.07 | 29.94 ± 6.47 | 26.07 ± 4.82 | 19.06 ± 3.64 |
|  | 31 | 29.04 ± 3.14 | 26.03 ± 3.04 | 24.21 ± 7.5 | 17.69 ± 6.32 |
|  | 310 | 27.73 ± 2.85 | 21.19 ± 4.65 | 28.36 ± 1.34 | 21.34 ± 1.37 |

Table S4: Outcome of the PERMANOVA on FA composition

|  | DF | SS | R^2^ | F-value | p-value |
| --- | --- | --- | --- | --- | --- |
| Origin | 1 | 1.084 | 0.512 | 41.968 | < 0.001 |
| Treatment | 3 | 0.167 | 0.080 | 2.159 | 0.045 |
| Origin:Treatment | 3 | 0.064 | 0.030 | 0.820 | 0.580 |
| Residual | 31 | 0.801 | 0.378 |  |  |
| Total | 38 | 2.116 | 1.000 |  |  |

Table S5: Bayesian factors for FA groups

| Origin | Ciprofloxacin treatment | SAFA | MUFA | PUFA | Omega 3 |
| --- | --- | --- | --- | --- | --- |
| Upstream-sourced | 3 | 5.18 | 1.68 | 3.65 | 2.08 |
|  | 31 | 9.60 | 12.6 | 10.4 | 1.18 |
|  | 310 | 16.8 | 99 | 1.10 | 7.21 |
| Downstream-sourced | 3 | 1.04 | 1.07 | 1.54 | 3.28 |
|  | 31 | 1.23 | 1 | 1.08 | 7.65 |
|  | 310 | 3.46 | 1.31 | 4.32 | 1.39 |
